# SETMAR Facilitates the Differentiation of Thyroid Cancer by Regulating SMARCA2‐Mediated Chromatin Remodeling

**DOI:** 10.1002/advs.202401712

**Published:** 2024-06-20

**Authors:** Wei Zhang, Xianhui Ruan, Yue Huang, Weiyu Zhang, Guangwei Xu, Jingzhu Zhao, Jie Hao, Nan Qin, Jinjian Liu, Qian Su, Jianfeng Liu, Mei Tao, Yuqi Wang, Songfeng Wei, Xiangqian Zheng, Ming Gao

**Affiliations:** ^1^ School of Medicine Nankai University 300000 Tianjin P. R. China; ^2^ Department of Thyroid and Neck Tumor Tianjin Medical University Cancer Institute and Hospital National Clinical Research Center for Cancer Key Laboratory of Cancer Prevention and Therapy Tianjin's Clinical Research Center for Cancer Huanhuxi Road, Ti‐Yuan‐Bei, Hexi District Tianjin 300060 P. R. China; ^3^ Department of Thyroid and Breast Surgery Tianjin Union Medical Center Tianjin 300131 P. R. China; ^4^ Tianjin Key Laboratory of General Surgery in Construction Tianjin Union Medical Center Tianjin 300131 P. R. China; ^5^ Department of Molecular Biology and Genetics Cornell University Ithaca NY 14851 USA; ^6^ School of Pharmacy Tianjin Medical University Tianjin Key Laboratory on Technologies Enabling Development Clinical Therapeutics and Diagnostics (Theragnostic) Tianjin 300000 P. R. China; ^7^ Key Laboratory of Radiopharmacokinetics for Innovative Drugs Chinese Academy of Medical Sciences Tianjin Key Laboratory of Radiation Medicine and Molecular Nuclear Medicine Institute of Radiation Medicine Chinese Academy of Medical Sciences & Peking Union Medical College Tianjin 300060 P. R. China; ^8^ Department of Molecular Imaging and Nuclear Medicine Tianjin Medical University Cancer Institute and Hospital National Clinical Research Center for Cancer Tianjin Key Laboratory of Cancer Prevention and Therapy Tianjin's Clinical Research Center for China Tianjin 300000 P. R. China

**Keywords:** chromatin remodeling, differentiation therapy, histone methylation, RNA M6A methylation, Thyroid cancer

## Abstract

Thyroid cancer is the most common type of endocrine cancer, and most patients have a good prognosis. However, the thyroid cancer differentiation status strongly affects patient response to conventional treatment and prognosis. Therefore, exploring the molecular mechanisms that influence the differentiation of thyroid cancer is very important for understanding the progression of this disease and improving therapeutic options. In this study, SETMAR as a key gene that affects thyroid cancer differentiation is identified. SETMAR significantly regulates the proliferation, epithelial‐mesenchymal transformation (EMT), thyroid differentiation‐related gene expression, radioactive iodine uptake, and sensitivity to MAPK inhibitor‐based redifferentiation therapies of thyroid cancer cells. Mechanistically, SETMAR methylates dimethylated H3K36 in the SMARCA2 promoter region to promote SMARCA2 transcription. SMARCA2 can bind to enhancers of the thyroid differentiation transcription factors (TTFs) PAX8, and FOXE1 to promote their expression by enhancing chromatin accessibility. Moreover, METTL3‐mediated m6A methylation of SETAMR mRNA is observed and showed that this medication can affect SETMAR expression in an IGF2BP3‐dependent manner. Finally, the METTL3‐14‐WTAP activator effectively facilitates the redifferentiation of thyroid cancer cells via the SETMAR‐SMARCA2‐TTF axis utilized. The research provides novel insights into the molecular mechanisms underlying thyroid cancer dedifferentiation and provides a new approach for therapeutically promoting redifferentiation.

## Graphical abstract

METTL3‐mediated m6A modification on SETMAR mRNA increases its stability in an IGF2BP3‐dependent manner. SETMAR methylates dimethylated H3K36 in the SMARCA2 promoter region to promote SMARCA2 transcription. SMARCA2 can bind to enhancers of the thyroid differentiation transcription factors (TTFs) PAX8, and FOXE1 to promote their expression by enhancing chromatin accessibility. The METTL3‐14‐WTAP activator can restore SETMAR expression and promote differentiation of thyroid cancer, thus providing therapeutic benefits.

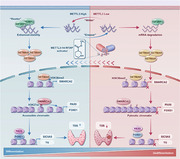

## 1 Introduction

Thyroid cancer is the most common type of endocrine cancer, and its incidence is rapidly increasing worldwide.^[^ ^1^, ^2^ ^]^ As tumor mutation load increases, well‐differentiated papillary thyroid carcinoma (PTC) can progress to poorly differentiated thyroid carcinoma (PDTC), and eventually to anaplastic thyroid carcinoma (ATC). Although the prognosis of PTC is generally good, with a ten‐year survival rate ranging from 80% to 95%, the prognosis of patients with ATC is extremely poor. These patients have a median survival time of less than 6 months, and ATC is the primary cause of thyroid cancer‐related death.^[^ ^3^ ^]^ PDTC and ATC have increased proliferation potential and exhibit characteristics of EMT. These types of tumors are also resistant to conventional treatments, including surgical and radioactive iodine treatment, leading to increased aggressiveness and lethality.^[^ ^4^ ^]^ Thyroid transcription factors, including PAX8, NKX2‐1, and FOXE1, control the expression of genes related to thyroid hormone biosynthesis and thyroid development.^[^ ^5^, ^6^ ^]^ However, in thyroid cancer, especially in the dedifferentiated subtype, oncoproteins that activate MAPK signaling inhibit the expression of thyroid transcription factors and their targets, rendering these tumors resistant to radioactive iodine treatment. Therefore, a more profound understanding of the molecular mechanisms underlying thyroid cancer dedifferentiation is crucial for developing more effective therapeutic strategies to treat this disease.

Epigenetics is a crucial field of research related to tumor pathogenesis, and epigenetics research has revealed many innovative treatment strategies.^[^ ^7^, ^8^, ^9^ ^]^ Histone methylation, an extensively studied epigenetic modification, significantly influences the occurrence and progression of tumors. Histone methylation, which primarily occurs at histone H3 and histone H4, is dynamically regulated by methyltransferases and demethylases.^[^ ^10^ ^]^ Histone methylation‐related genes have been found to regulate the differentiation of cancer cells in colorectal cancer and acute myeloid leukemia. In colorectal cancer, KDM6A and KDM6B have been identified as inhibitors of colorectal cancer cell differentiation, and their overexpression is closely related to poor clinical prognosis in colorectal cancer patients.^[^ ^11^ ^]^ In acute myeloid leukemia, high expression of KDM1A can inhibit the myeloid differentiation of tumors and enhance their stem cell properties, thereby promoting cancer progression.^[^ ^12^ ^]^ Moreover, targeted inhibition of KDM1A has shown promising results in promoting myeloid differentiation and has good therapeutic benefits.^[^ ^13^ ^]^ Despite their significance in cancer differentiation, the role of histone methylation modifiers in thyroid cancer differentiation is poorly understood and requires further investigation.

The histone methyltransferase SETMAR is a fusion protein that contains an N‐terminal histone lysine N‐methyltransferase domain and a C‐terminal transposase domain.^[^ ^14^ ^]^ It has been reported that SETMAR is involved in the catalytic methylation of histone H3K4me2, H3K36me2, and H3K27me3 sites, thus participating in regulating gene expression.^[^ ^15^ ^]^ Several studies have reported that SETMAR performs different functions in tumors, depending on genetic background^[^ ^16^, ^17^, ^18^ ^]^; thus, SETMAR is very important in both cancer development and treatment. However, the role of SETMAR in regulating cancer cell differentiation has not been previously investigated.

In the present study, we first identified SETMAR as an activator that promotes the differentiation of thyroid cancer cells, and its expression level is markedly decreased with increasing thyroid dedifferentiation. In vivo and in vitro experiments proved that SETMAR can significantly facilitate the transcription of TTFs through the induction of SMARCA2‐mediated chromatin remodeling to affect the proliferation, EMT, RAI treatment uptake capacity, and sensitivity to MAPK inhibitor‐based redifferentiation therapies of thyroid cancer cells. In addition, we found that the dysregulation of SETMAR during thyroid cancer dedifferentiation is caused by a lack of METTL3‐mediated N6‐methyladenosine modification which maintains the stability of SETMAR mRNA. More importantly, we utilized the METTL3‐14‐WTAP activator to restore SETMAR expression and promote the differentiation of thyroid cancer, thus providing a new method of redifferentiation therapy for treating patients with thyroid cancer.

## 2 Results

### 2.1 SETMAR was Identified as a Histone Methylation Modifier that is Involved in Thyroid Cancer Differentiation

**Figure 1:**
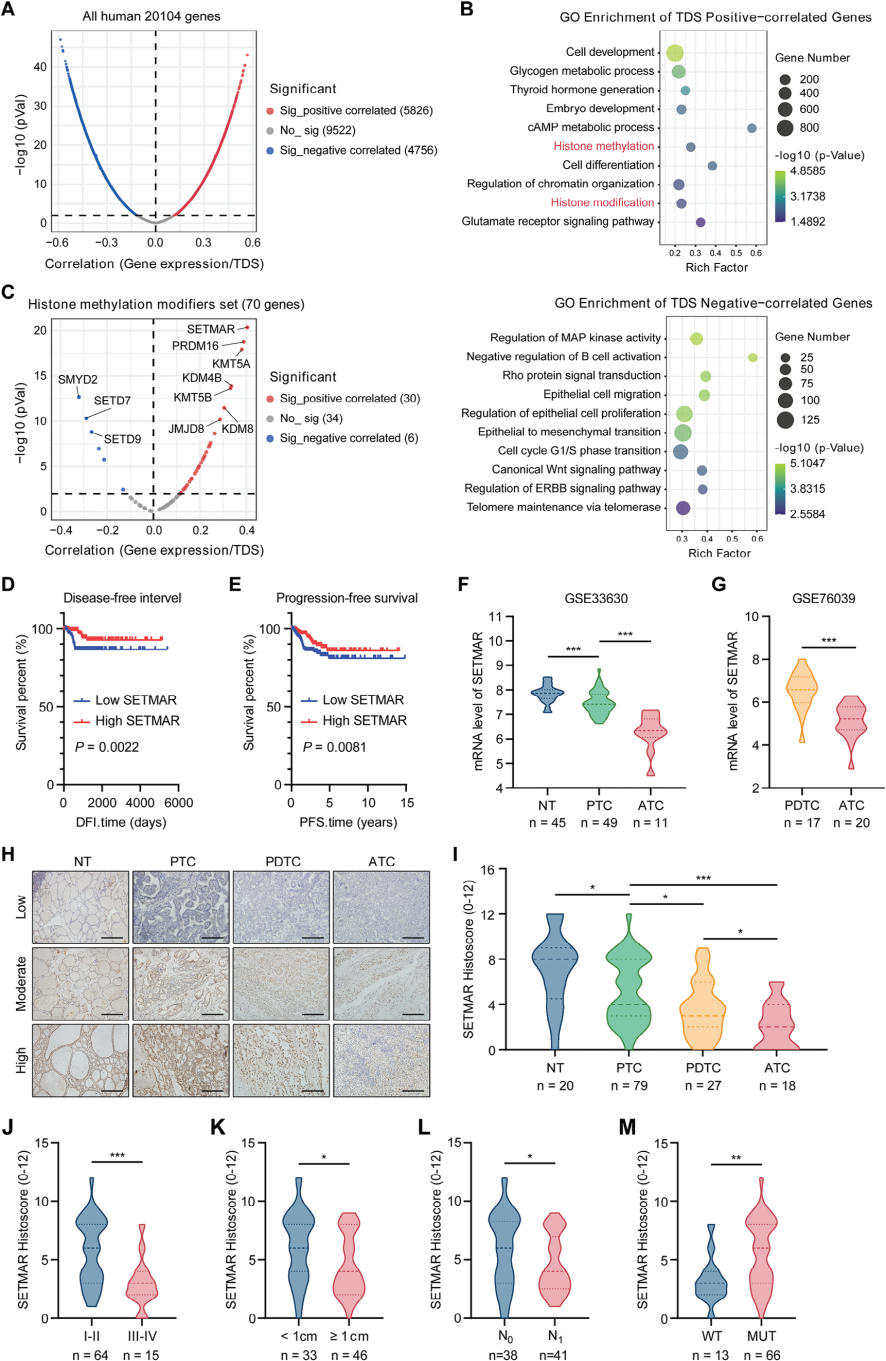
SETMAR was identified as a histone methylation modifier that is involved in thyroid cancer differentiation. A) A volcano plot was generated to show the correlation between genome‐wide gene expression and TDSs in the TCGA database. B) GO analysis was performed on genes that showed significant positive or negative correlations with TDSs in the TCGA database. C) Another volcano plot was created to demonstrate the correlation between histone methylation gene expression and TDSs in the TCGA database. D,E) Kaplan–Meier survival analysis showed that low SETMAR expression was significantly associated with poor patient disease‐free survival (D) and poor progression‐free survival (E) in the TCGA database (*P* values were determined by log‐rank test). F,G) Analysis of SETMAR expression in different types of thyroid carcinoma and normal thyroid tissue in the GSE33630 (F) and GSE70639 (G) databases. H,I) Immunohistochemical staining for SETMAR was performed in normal thyroid (NT) tissue, papillary thyroid carcinoma (PTC) tissue, poorly differentiated thyroid carcinoma (PDTC) tissue, and anaplastic thyroid carcinoma (ATC) tissue. The differences in SETMAR expression of various cancer subtypes were also analyzed. J,M) Correlation analysis of the immunohistochemical staining for SETMAR with patient clinical stage (J), tumor size (K), lymph node metastasis (L), and BRAF mutation (M) in patients with papillary thyroid carcinoma. Data are shown as the median with a range in (F, G, I, J, K, L, and M). *p* values were determined using a two‐tailed unpaired Student's *t*‐test (^*^ *p* < 0.05, ^**^ *p* < 0.01, ^***^ *p* < 0.001).

To identify pivotal gene signatures involved in regulating thyroid cancer differentiation, we utilized the Thyroid Differentiation Score (TDS), which summarizes the expression levels of 16 genes associated with thyroid metabolism and function,^[^ ^19^ ^]^ to assess the degree of tumor differentiation in individuals thyroid cancer patients in the TCGA database. We conducted an analysis of the correlation between human genome‐wide gene expression and TDSs in the TCGA database (**Figure** **1**A) and found that a total of 5826 genes were significantly positively correlated with the TDS, while 4756 genes were significantly negatively correlated with the TDS. We subsequently performed Gene Ontology (GO) analysis on the top 3000 genes that exhibited significant positive or negative correlations with the TDS. The results showed that histone modification, especially methylation, may be an important biological process that affects the differentiation of thyroid cancer (Figure 1B). Thus, we examined the relationship between the expression of 70 histone modification‐related genes and the TDS in patients from the TCGA database. Our analysis revealed that most genes in this gene family were positively correlated with TDSs; among these genes, SETMAR expression was most strongly correlated with TDSs (Figure 1C).

According to the TCGA database analysis, we observed a significant positive correlation between the expression of SETMAR and most TDS‐related genes (Figure S1A, Supporting Information). SETMAR expression was significantly lower in classic papillary thyroid carcinomas (CPTCs) than in normal tissues, and high‐cell variant PTCs, which are associated with more aggressive clinical behavior, exhibited even lower levels of SETMAR expression than CPTCs (Figure S1B, Supporting Information). Correspondingly, the expression level of SETMAR was significantly associated with various clinical characteristics of thyroid cancer, including clinical stage (Figure S1C, Supporting Information), T stage (Figure S1D, Supporting Information), lymph node metastasis (Figure S1E, Supporting Information), BRAF^V600E^ mutation (Figure S1F, Supporting Information), disease‐free survival (Figure 1D), and progression‐free survival (Figure 1E). Additionally, when examining different GEO datasets, including GSE33630 and GSE76039, we noticed a trend toward low SETMAR expression in poorly differentiated thyroid cancer (Figure 1F,G).

Subsequently, we examined SETMAR expression patterns in the clinical samples we collected. First, we examined the mRNA and protein expression levels of SETMAR in randomly selected PTC tissues and their corresponding adjacent normal tissues, and the results showed that the expression of SETMAR in PTC tissues was significantly lower than in normal tissues (Figure S1G,H, Supporting Information). Next, we measured SETMAR protein expression in normal thyroid (20 cases), PTC (79 cases), PDTC (27 cases), and ATC (18 cases) staining by IHC staining (Figure 1H). The results indicated that the expression of SETMAR in tumor tissues was significantly lower than that in normal tissues, and its expression level was notably lower in relatively poorly differentiated tumors (Figure 1I). Moreover, in the PTC cohort, SETMAR expression was correlated with clinical stage, tumor size, lymph node metastasis, and BRAF^V600E^ mutation (Figure 1J–M). We also measured the expression of SETMAR in human normal thyroid, PTC, and ATC cell lines. The results showed that the expression of SETMAR in thyroid cancer cells was lower than that in normal thyroid cells, especially in ATC cells. (Figure S1I,J, Supporting Information).

**Figure 2:**
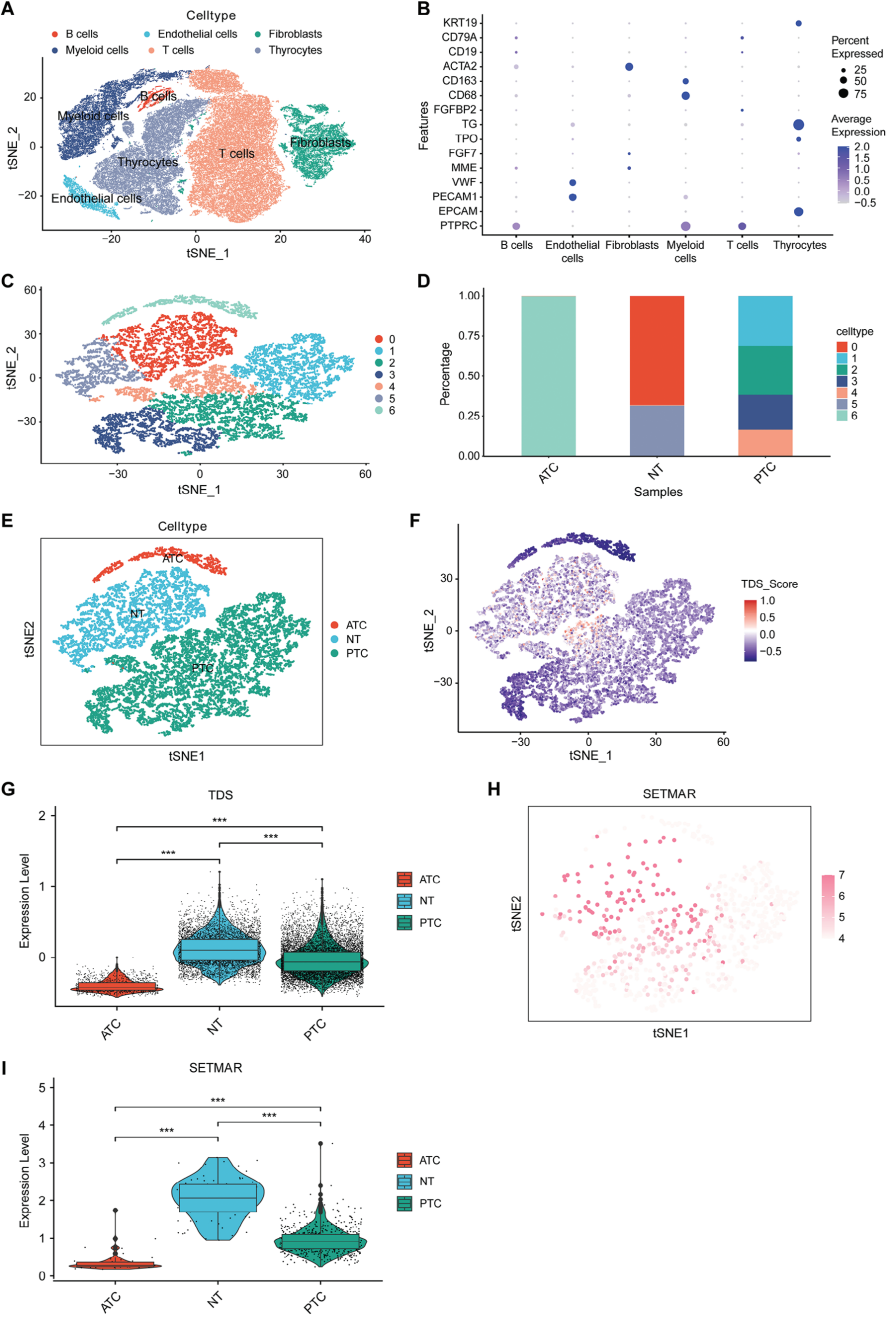
Investigation of the effect of SETMAR expression on thyroid cancer differentiation in single‐cell datasets. A) A TSNE plot displaying the clustering of single‐cell data from 10 samples into 6 distinct groups. B) Bubble plot showing the expression of marker genes in cells from different groups. C) TSNE plot of 7 groups of thyroid cells (normal + tumor) obtained by dimensionality reduction clustering. D) The proportion of cell clusters in each sample. E) Dimensionality reduction clustering results for thyroid follicular cells and tumor cells. F,G) Verification of the grouping of thyroid follicular cells and tumor cells by examining differences in TDSs. H,I) Verification of differences in SETMAR expression in thyroid follicular cells, PTC cells, and ATC cells. Data are shown as the median with a range in (G, I). *p* values were determined using a two‐tailed unpaired Student's *t*‐test (^***^ *p* < 0.001).

Finally, we conducted an analysis using single‐cell sequencing datasets to investigate the impact of SETMAR expression on tumor differentiation at the single‐cell level. We integrated several thyroid cancer single‐cell sequencing datasets, including GSE148673, GSE184362, and GSE134355. After integrating and performing dimensionality reduction clustering, we identified various cell groups, including normal thyroid cells, PTC cells, and ATC cells (**Figure** **2**A–E). Furthermore, we specifically analyzed thyroid follicular cells and tumor cells to determine the differences in TDSs and the expression of SETMAR in these groups of cells. The results indicated that the TDS level and SETMAR expression were lower in tumor cells, particularly in ATC cells (Figure 2F–I).

In summary, these results demonstrated the crucial role of SETMAR in regulating the differentiation of thyroid cancer. Thus, SETMAR is a promising therapeutic target and prognostic marker for thyroid cancer.

### 2.2 SETMAR Facilitates the Differentiation of Thyroid Cancer In Vitro and In Vivo

**Figure 3:**
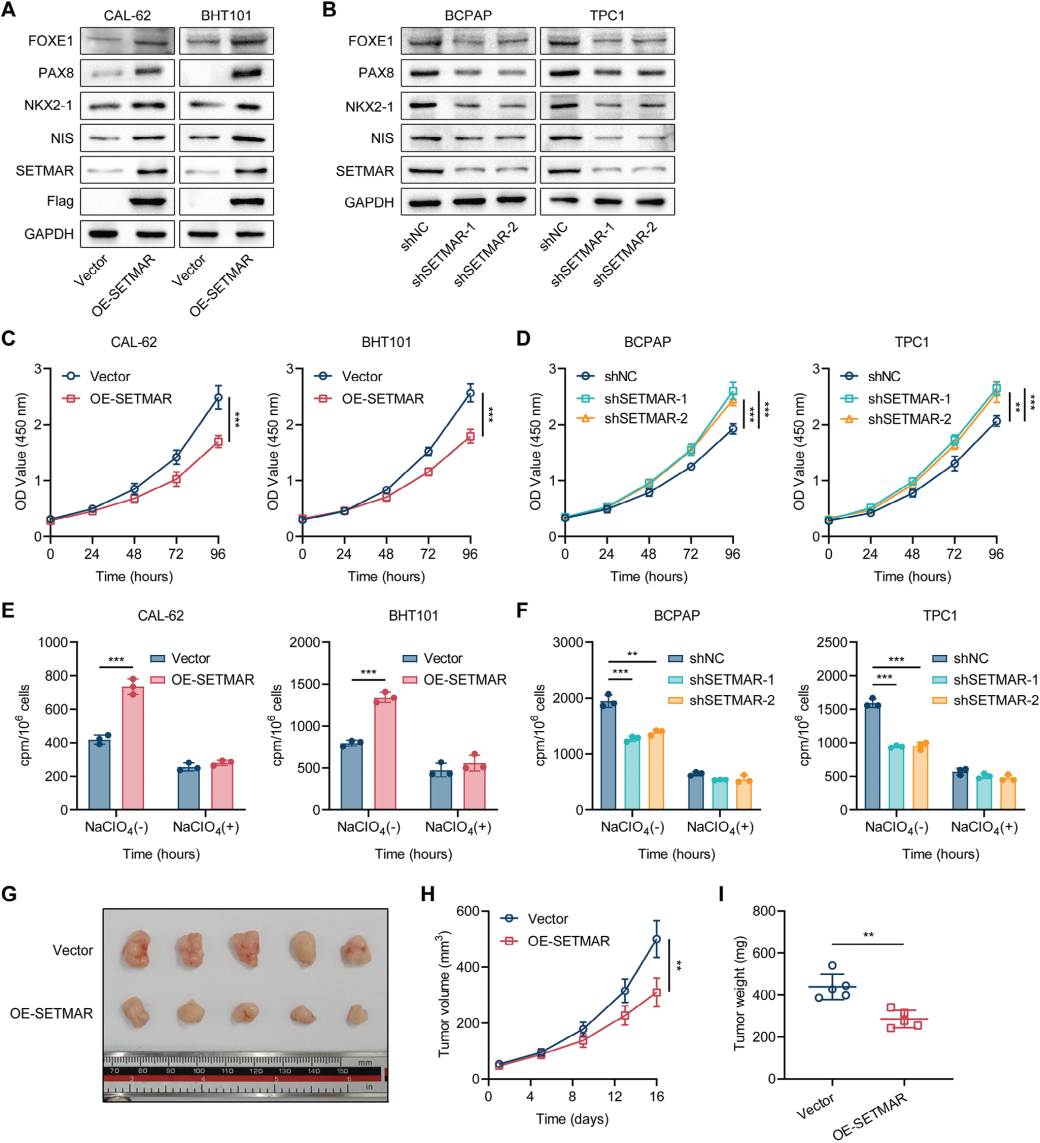
SETMAR facilitates the differentiation of thyroid cancer in vitro and in vivo. A,B) Western blotting was used to analyze the effects of SETMAR overexpression (A) or knockdown (B) on the expression of thyroid cancer cell differentiation markers, including PAX8, FOXE1, NKX2‐1, and NIS. C, D) A CCK‐8 assay was used to assess the impact of SETMAR overexpression (A) or knockdown (B) on the proliferation of thyroid cancer cells. E,F) A radioactive iodine uptake assay was used to determine the influence of SETMAR overexpression (E) or knockdown (F) on the ability of thyroid cancer cells to take up radioactive iodine. G) Representative images of subcutaneous xenografts in nude mice derived from SETMAR‐overexpressing or control CAL‐62 cells (*n* = 5 for each group). H) Growth curves of the subcutaneous xenografts in the SETMAR‐overexpressing group and control group. I) Analysis of the tumor weights of xenografts in the SETMAR‐overexpressing group and control group. Data are shown as the mean ± SD of three replicates in (C, D, E, and F) and five replicates in (H, I). *P* values were determined using a two‐tailed unpaired Student's *t*‐test (^*^ *p* < 0.05, ^**^ *p* < 0.01, ^***^ *p* < 0.001).

To further investigate the regulatory effect of SETMAR on the differentiation of thyroid cancer, we overexpressed SETMAR in two ATC cell lines (CAL‐62 and BHT101) that had relatively low levels of endogenous SETMAR expression (**Figure** **3**A). Additionally, we utilized two independent shRNAs to inhibit the expression of SETMAR in two PTC cell lines (BCPAP and TPC1) that had higher levels of endogenous SETMAR expression (Figure 3B). The results revealed that overexpression of SETMAR in vitro effectively enhanced the expression of differentiation markers in thyroid cancer cells (Figure 3A; Figure S2A, Supporting Information), leading to inhibition of thyroid cancer proliferation (Figure 3C) and increased radioactive iodine uptake (Figure 3E). In contrast, the knockdown of SETMAR in vitro significantly reduced the expression of differentiation markers in thyroid cancer cells (Figure 3B; Figure S2B, Supporting Information), increased proliferation (Figure 3D) and decreased radioactive iodine uptake (Figure 3F). We investigated the impact of SETMAR knockdown or overexpression on the global abundance of H3K4me2 and H3K36me2, which are well‐known histone methylation sites that are affected by SETMAR. Our findings revealed that only the global abundance of H3K36me2 was altered in thyroid cancer cells as the expression of SETMAR changed (Figure S2C,D, Supporting Information).

Considering the important impact of tumor differentiation on the EMT process,^[^ ^20^ ^]^ we measured the expression of EMT‐related markers in SETMAR‐knockdown or SETMAR‐overexpressing thyroid cancer cells. We observed that the expression of epithelial differentiation markers (E‐cadherin) increased, and the expression of mesenchymal differentiation markers (Snail, Slug, and N‐cadherin) decreased due to SETMAR overexpression (Figure S2C, Supporting Information). Conversely, SETMAR knockdown resulted in an increase in the expression of mesenchymal differentiation markers and a decrease in the expression of epithelial differentiation markers (Figure S2D, Supporting Information). Correspondingly, the overexpression of SETMAR reduced the migration and invasion of thyroid cancer cells (Figure S2E, Supporting Information), while SETMAR knockdown enhanced these abilities in tumor cells (Figure S2F, Supporting Information).

Increasing evidence suggests that impaired stemness and enhanced differentiation contribute to overcoming chemotherapy resistance in cancer cells.^[^ ^3^ ^]^ Therefore, we hypothesized that SETMAR might influence the sensitivity of thyroid cancer cells to chemotherapeutic drugs, such as doxorubicin (DOX) and paclitaxel (PTX). The overexpression of SETMAR in thyroid cancer cells increased their sensitivity to DOX treatment to a certain extent, whereas SETMAR knockdown reduced this sensitivity (Figure S3A, Supporting Information). The same phenomenon was observed in SETMAR‐knockdown or SETMAR‐overexpressing thyroid cancer cells that were treated with PTX (Figure S3B, Supporting Information).

To validate the ability of SETMAR to facilitate the differentiation of thyroid cancer cells in vivo, we established a tumor xenograft model by subcutaneously injecting CAL62 cells into nude mice (Figure 1G). Compared with those in the control group, xenografts formed from SETMAR‐overexpressing CAL‐62 cells exhibited weaker proliferation, as shown by decreased tumor volume and tumor weight (Figure 1H,I). Immunohistochemical staining of xenografts confirmed the overexpression of SETMAR in vivo and the resulting decreasing proliferation and increasing differentiation, as determined by staining for SETMAR, Ki67, PAX8, and NIS (Figure S3C, Supporting Information). Taken together, these findings suggest that SETMAR is involved in regulating various biological behaviors of thyroid cancer. SETMAR can facilitate the differentiation of thyroid cancer cells and exert a tumor suppressor effect.

### 2.3 SETMAR Altered the Expression of Genes Involved in Chromatin Remodeling

**Figure 4:**
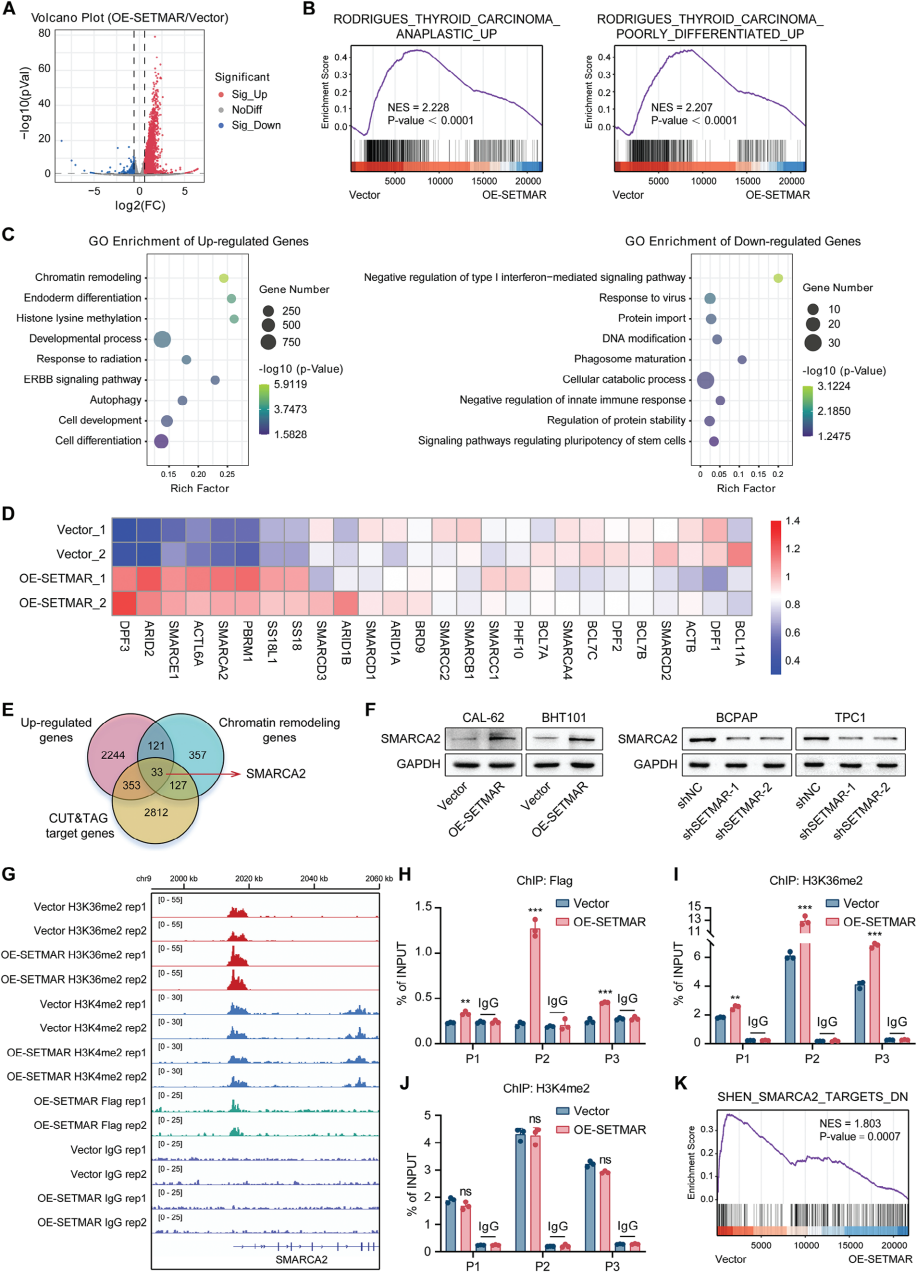
SETMAR altered the expression of genes involved in chromatin remodeling. A) Volcano plot of the RNA‐seq analysis of SETMAR‐overexpressing CAL‐62 cells. B) GSEA showed that the differentially expressed genes between SETMAR‐overexpressing cells and control cells were significantly enriched in gene sets related to thyroid dedifferentiation. C) Representative GO term analysis of upregulated genes (red) and downregulated genes (blue) after SETMAR overexpression. D) Heatmap from the RNA‐Seq data showing the gene expression of SWI/SNF subunits in SETMAR‐overexpressing or control CAL‐62 cells. E) Venn diagram demonstrating that 33 genes overlapped among genes that were overexpressed after SETMAR overexpression, as detected by RNA‐seq; target genes, as identified by CUT & Tag; and chromatin remodeling‐related genes. F) Western blotting was used to assess changes in SMARCA2 expression following SETMAR overexpression or knockdown. G) SETMAR, H3k36me2, and H3K4me2 occupancy in the vicinity of the SMARCA2 promoter was assessed by CUT & Tag in SETMAR‐3 × Flag‐overexpressing CAL‐62 cells. H,I,J) The enrichment levels of Flag (H), H3K36me2 (I), and H3K4me2 (J) in different regions (chr9:2015649–2013770, P1; chr9:2014750–2014823, P2; chr9:2015618–2015688, P3) of the SMARCA2 gene promoter in SETMAR‐3 × Flag‐overexpressing or control CAL‐62 cells were determined by ChIP‒qPCR. (K) GSEA confirmed that SETMAR promotes the expression of SMARCA2 targets. Data are shown as the mean ± SD of three replicates in (H, I, and J). *p* values were determined using two‐tailed unpaired Student's *t*‐test (^*^ *p* < 0.05, ^**^ *p* < 0.01, ^***^ *p* < 0.001. ns, no significance).

To investigate the effect of SETMAR on gene expression, we performed RNA sequencing of control cells and SETMAR‐overexpressing cells to identify differentially expressed genes. SETMAR overexpression upregulated 2751 genes and downregulated 228 genes (**Figure** **4**A). The GSEA results showed that overexpression of SETMAR can significantly promote the differentiation of thyroid cancer cells (Figure 4B). Then, GO enrichment analysis was performed on the differentially expressed genes. These findings indicated that SETMAR regulates several vital signaling pathways and biological processes related to development, differentiation, and stem cell pluripotency, indicating that SETMAR plays essential roles in the differentiation of thyroid cancer (Figure 4C). Moreover, the genes whose expression was up‐regulated after SETMAR overexpression were significantly enriched in chromatin remodeling (Figure 4C). A previous study reported that loss of function of the SWI/SNF complex due to mutations in its subunits promotes the progression of thyroid tumors and renders thyroid tumors resistant to redifferentiation therapies.^[^ ^21^ ^]^ After analyzing the RNA‐seq results, it was observed that the expression of several subunits of the SWI/SNF complex was increased following SETMAR overexpression (Figure 4D). Based on these findings, we speculated that SETMAR may enhance the differentiation of thyroid cancer by activating the function of the SWI/SNF complex.

To investigate the mechanism by which SETMAR regulates gene expression, we performed CUT&Tag experiments to determine the genome‐wide target sites of SETMAR in thyroid cancer cells. We identified 4362 peaks corresponding to 3325 RefSeq genes (Figure S4A, Supporting Information). The enrichment of H3K36me2 in SETMAR overexpressing cells was significantly greater than in control cells, while there was a minimal difference in H3K4me2 levels between the two groups (Figure S4B, Supporting Information). We subsequently identified 33 crucial genes by overlapping the genes that were upregulated after SETMAR overexpression, SETMAR‐targeted genes identified by CUT&Tag, and genes related to chromatin remodeling. Interestingly, we found that SMARCA2 was included in this set of 33 genes (Figure 4E); this gene belongs to the ATPase subunits of the SWI/SNF complex and is the core element that supplies energy for SWI/SNF complex‐mediated chromatin remodeling.^[^ ^22^ ^]^ Therefore, we wanted to determine whether SETMAR facilitates thyroid cancer differentiation through SMARCA2‐activated chromatin remodeling.

First, we verified that SETMAR has a regulatory effect on SMARCA2. We found that overexpression of SETMAR could promote the protein and mRNA expression of SMARCA2 in thyroid cancer cells, while inhibition of SETMAR had the opposite effect (Figure 4F; Figure S4C, Supporting Information). The CUT&Tag assay also confirmed that SETMAR bound to the promoter region of SMARCA2 and augmented H3K36me2 enrichment in this genomic region, but the enrichment of H3K4me2, another methylation site, did not change (Figure 4G). Primers spanning the proximal promoter regions of SMARCA2 were designed for ChIP‐qPCR. The results demonstrated that SETMAR is bound to the promoter region of SMARCA2 (Figure 4H), resulting in an augmented enrichment of H3K36me2 in this specific region (Figure 4I). However, it did not have any impact on the enrichment of H3K4me2 (Figure 4J). Moreover, GSEA showed that SETMAR overexpression promoted the expression of SMARCA2 target genes (Figure 4K). In summary, we found that SETMAR can control the expression of genes related to chromatin remodeling and that SMARCA2 is a direct target of SETMAR.

### 2.4 SETMAR Promotes Thyroid Cancer Differentiation via its Regulation of SMARCA2 Through its Methyltransferase Activity

**Figure 5:**
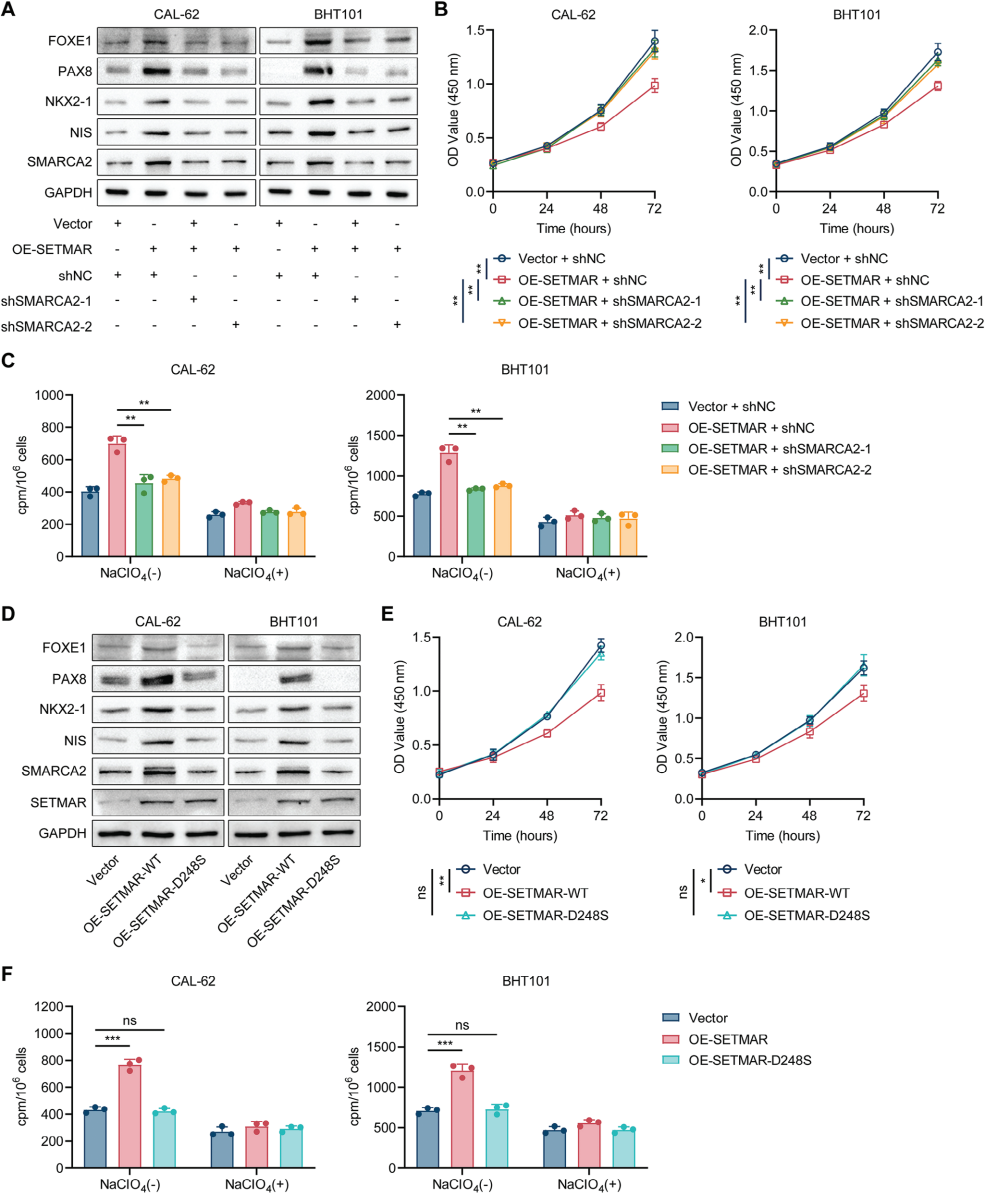
SETMAR promotes thyroid cancer differentiation by regulating SMARCA2 through its methyltransferase activity. A) Western blotting confirmed that shRNA‐mediated inhibition of SMARCA2 expression could reverse the SETMAR overexpression‐induced increase in thyroid differentiation marker expression in ATC cells. B) CCK‐8 assays demonstrated that the inhibition of ATC cell proliferation caused by SETMAR overexpression could be reversed by SMARCA2 silencing. C) Knocking down SMARCA2 in ATC cells overexpressing SETMAR reversed the SETMAR overexpression‐induced increase in radioactive iodine uptake. D) Western blotting was used to determine the effect of methyltransferase‐deficient SETMAR overexpression on the expression of thyroid differentiation markers and SMARCA2 compared with wild‐type SETMAR overexpression in ATC cells. E) A CCK8 assay was used to detect the effect of overexpressing wild‐type SETMAR or SETMAR‐D248S on the proliferation of ATC cells. F) A radioactive iodine uptake assay was performed to evaluate the effect of overexpressing wild‐type SETMAR or SETMAR‐D248S on the radioactive iodine uptake of ATC cells. Data are shown as the mean ± SD of three replicates in (B, C, E, and F). *p* values were determined using two‐tailed unpaired Student's *t*‐test (^*^ *p* < 0.05, ^**^ *p* < 0.01, ^***^ *p* < 0.001. ns, no significance).

To confirm that SETMAR promotes thyroid cancer differentiation by regulating SMARCA2, we transfected shRNAs against SMARCA2 into SETMAR‐overexpressing ATC cells. The results showed that inhibition of SMARCA2 reversed the changes in phenotype that were caused by SETMAR overexpression in ATC cells, which included upregulated protein expression levels of thyroid differentiation markers (**Figure** **5**A), impaired proliferation (Figure 5B), and the enhanced radioactive iodine uptake (Figure 5C).

To further assess the role of SETMAR methyltransferase activity in thyroid cancer differentiation, we overexpressed a methyltransferase‐deficient mutant of SETMAR, SETMAR‐D248S,^[^ ^23^ ^]^ in ATC cells. Compared to overexpression of wild‐type SETMAR, SETMAR‐D248S overexpression failed to promote SMARCA2 and thyroid differentiation markers’ expression (Figure 5D; Figure S4D, Supporting Information), inhibit proliferation (Figure 5E), or increase radioactive iodine uptake (Figure 5F) in ATC cells. Moreover, overexpression of SETMAR‐D248S also could not change the enrichment level of H3K36me2 in SEMARCA2 promoter region (Figure S4E, Supporting Information). These findings suggested that the ability of SETMAR to support thyroid carcinoma differentiation depends on its methyltransferase activity.

### 2.5 SMARCA2 Sustains Thyroid Cancer Differentiation by Mediating Chromatin Remodeling

To further explore how SETMAR‐regulated SMARCA2 plays a role in promoting differentiation in thyroid cancer, we employed the CRISPRa technique to overexpress SMARCA2 in ATC cells by activating its endogenous promoter. The results indicated that SMARCA2 overexpression substantially enhanced the expression of thyroid differentiation markers at both the protein and mRNA levels (**Figure** **6**A; Figure S5A, Supporting Information). SMARCA2 was also found to inhibit proliferation, promote radioactive iodine uptake, and weaken the migration and invasion of ATC cells in vitro (Figure S5B–D, Supporting Information). Additionally, SMARCA2 also attenuated the proliferative potential of thyroid cancer cells in vivo (Figure S6A–C, Supporting Information). Immunohistochemical staining of xenografts confirmed that in vivo overexpression of SMARCA2 increased the expression of thyroid differentiation markers and suppressed the proliferation‐related marker Ki67 (Figure S6D, Supporting Information).

Figure 6SMARCA2 sustains thyroid cancer differentiation by mediating chromatin remodeling. A) After the overexpression of SMARCA2 in ATC cells using the CRISPR/Cas9a technique, Western blotting analysis was conducted to confirm the impact of SMARCA2 overexpression on thyroid differentiation marker expression. B) Tornado plots of ATAC‐seq signals showing the ATAC‐seq peak gains and losses after SMARCA2 overexpression. C) Transcription factor motifs enriched in ATAC‐seq peak gains (red) and losses (blue) identified using HOMER de novo motif discovery. D) ChIP‐seq of H3K27Ac and SMARCA2 and ATAC‐seq were used to detect changes in chromatin structure near the PAX8 and FOXE1 loci in SMARCA2‐overexpressing and control CAL‐62 cells. Track heights were normalized to the relative number of mapped reads. E) ChIP‒qPCR was performed to confirm that SMARCA2 bound to the putative enhancers of PAX8 (chr2: 114041886–114041992) and FOXE1 (chr9: 100639091–100639219) and increased H3K27Ac enrichment. F) The GeneHancer database was used to verify that enhancers that bound to SMARCA2 interacted with the promoters of PAX8 and FOXE1. G) Chromosome conformation capture (3C) assay was performed to verify that enhancers that bound to SMARCA2 interacted with the promoters of PAX8 and FOXE1. H, I) Verification of differences in SMARCA2 expression in thyroid follicular cells, PTC cells, and ATC cells in the integrated single‐cell sequencing dataset. Data are shown as the mean ± SD of three replicates in (E) and the median with range in (I). *p* values were determined using a two‐tailed unpaired Student's *t*‐test (^**^ *p* < 0.01, ^***^ *p* < 0.001. ns, no significance).

**Figure:**
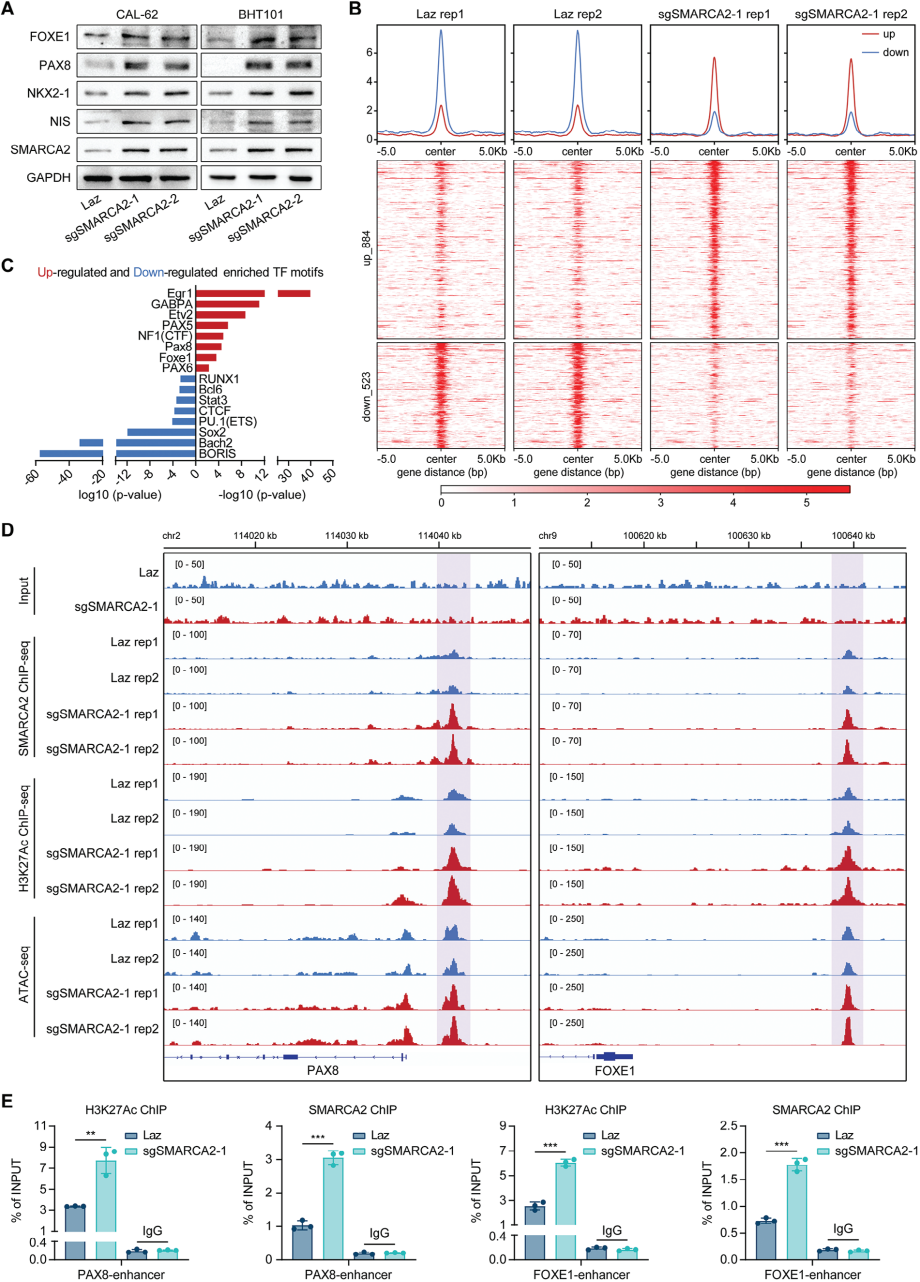

**Figure:**
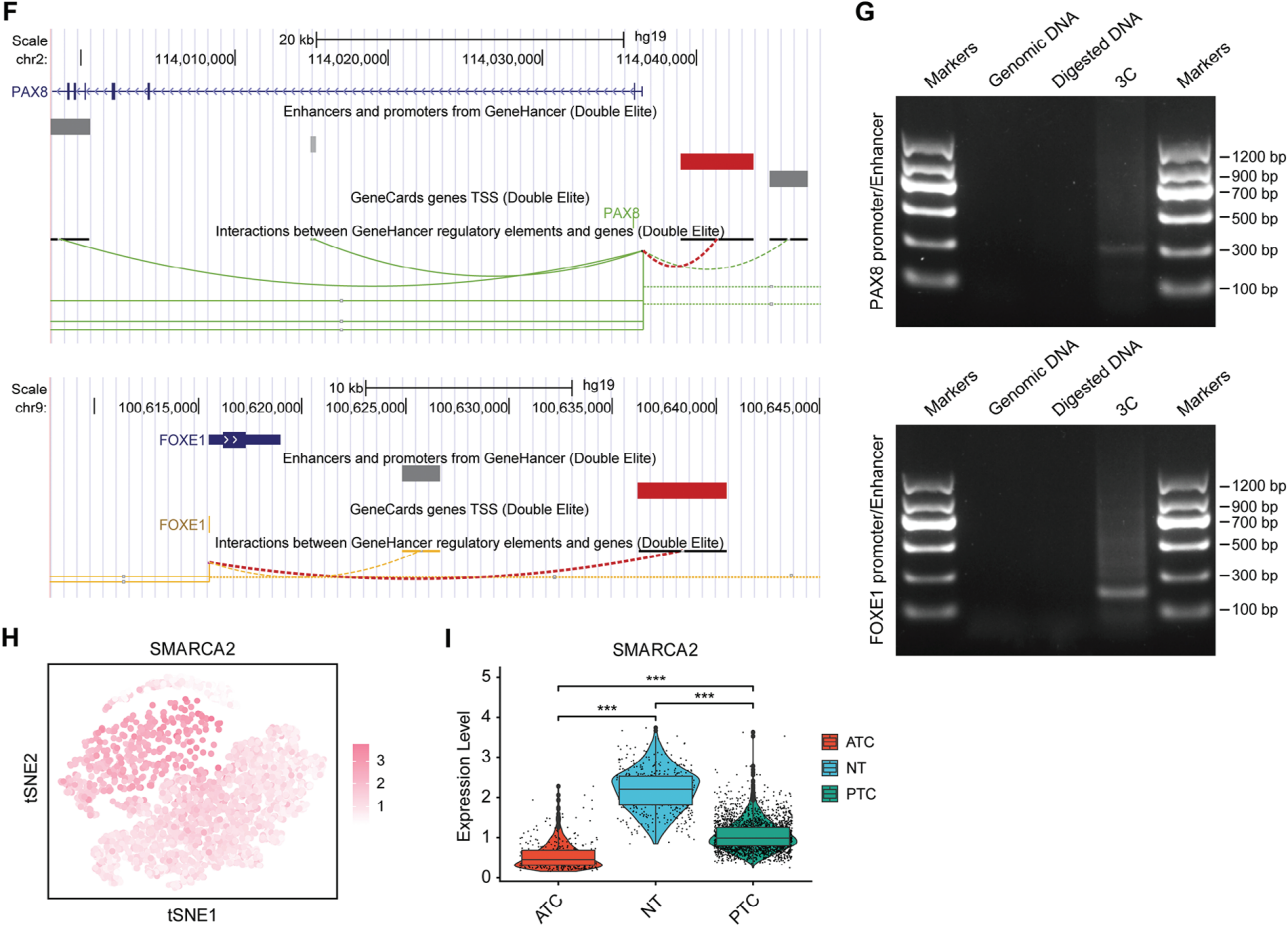

To investigate the impact of SMARCA2 on genome‐wide chromatin accessibility we performed ATAC‐Seq on SMARCA2‐overexpressing or control ATC cells. Overexpression of SMARCA2 led to 884 upregulated ATAC peaks and 523 downregulated ATAC peaks compared to those in control cells (Figure 6B). Using HOMER de novo motif discovery, we identified transcription factor motifs that were enriched in both the upregulated and downregulated ATAC peaks (Figure 6C). After SETMAR overexpression, the accessibility of the Foxe1 and Pax8 DNA binding sites was significantly increased (Figure 6C). The same occurred with NF1/CTF, which forms a complex with the pioneer transcription factor FOXE1 of the thyroid lineage (Figure 6C).

Considering the chromatin remodeling effect of SWI/SNF, we focused on studying the chromatin structure of the PAX8 and FOXE1 loci and their possible regulation by SMARCA2. Indeed, in ChIP‐seq data from CAL‐62 cells, increased SMARCA2 occupancy was observed at adjacent sites in the PAX8 and FOXE1 promoters when SMARCA2 was overexpressed (Figure 6D). Consistent with this, transposase‐accessible chromatin analysis using ATAC peaks was also increased in these genomic regions following SMARCA2 overexpression (Figure 6D). Since the recruitment of SWI/SNF complexes to enhancer or promoter regions is a common occurrence in the regulation of gene transcription,^[^ ^24^, ^25^ ^]^ we also examined the ChIP‐seq signal of H3K27Ac, which is a chromatin mark associated with active enhancers, around PAX8 and FOXE1. We found that there were increased H3K27Ac ChIP‐seq signals in putative enhancer regions of PAX8 and FOXE1 in ATC cells overexpressing SMARCA2 (Figure 6D). We designed primers spanning the binding regions of SMARCA2 in putative enhancers of PAX8 and FOXE1 and performed ChIP–qPCR to confirm that SMARCA2 can bind to these regions and increase H3K27Ac enrichment (Figure 6E). Moreover, according to the investigation of the GeneHancer database, the enhancers to which SMARCA2 was anchored are considered to interact with the promoters of PAX8 and FOXE1 (Figure 6F). We also performed a Chromosome conformation capture (3C) assay to verify the interactions between the enhancers to which SMARCA2 was anchored and the promoters of PAX8 and FOXE1 (Figure 6G). All these findings suggested that the SMARCA2‐containing SWI/SNF complex might attach to the enhancer region of important transcription factors of the thyroid lineage, such as PAX8 and FOXE1. These binding events increase the chromatin accessibility of PAX8 and FOXE1, which in turn increases the transcription of those genes.

Finally, we examined the expression pattern of SMARCA2 in thyroid cancer. Based on our analysis of the single‐cell dataset, we observed a significant decrease in the expression of SMARCA2 in tumor cells, particularly in anaplastic thyroid cancer (ATC) cells, compared to that in normal thyroid follicular epithelial cells (Figure 6H,I). Analysis of the TCGA database revealed a positive correlation between the expression of SMARCA2 and the TDS as well as between the expression of SMARCA2 and SETMAR (Figure S7A,B, Supporting Information). The expression of SMARCA2 in tumor tissues was lower than that in normal thyroid tissues, especially in high‐cell tumors (Figure S7C, Supporting Information). Moreover, according to the TCGA database, there were correlations between high SMARCA2 expression and longer progression‐free survival as well as longer disease‐free survival (Figure S7D,E, Supporting Information). By analyzing different GEO datasets, we also found that SMARCA2 expression was lower in poorly differentiated tumor types (Figure S7F,G, Supporting Information). Finally, we utilized immunohistochemical staining to detect the expression of SMARCA2 in normal tissues (19 cases), PTC tissues (51 cases), PDTC tissues (27 cases), and ATC tissues (18 cases) (Figure S7H, Supporting Information). Similar to the expression pattern of SETMAR, the expression of SMARCA2 was also reduced in relatively poorly differentiated tumor tissues (Figure S7I, Supporting Information). SMARCA2 expression was strongly associated with tumor stage (Figure S7J, Supporting Information), tumor size (Figure S7K, Supporting Information), and lymph node metastasis (Figure S7L, Supporting Information). However, there was no significant association between the expression level of SMARCA2 and the BRAF^V600E^ mutation status of the tumor (Figure S7M, Supporting Information). Immunohistochemical staining of NIS was also performed on the PTC tissues of this cohort. Our analysis of histoscores revealed a positive correlation among SETMAR, SMARCA2, and NIS (Figure S8A,B, Supporting Information).

### 2.6 METTL3‐Mediated m6A Modification Stabilizes SETMAR mRNA in an IGF2BP3‐Dependent Manner

**Figure 7:**
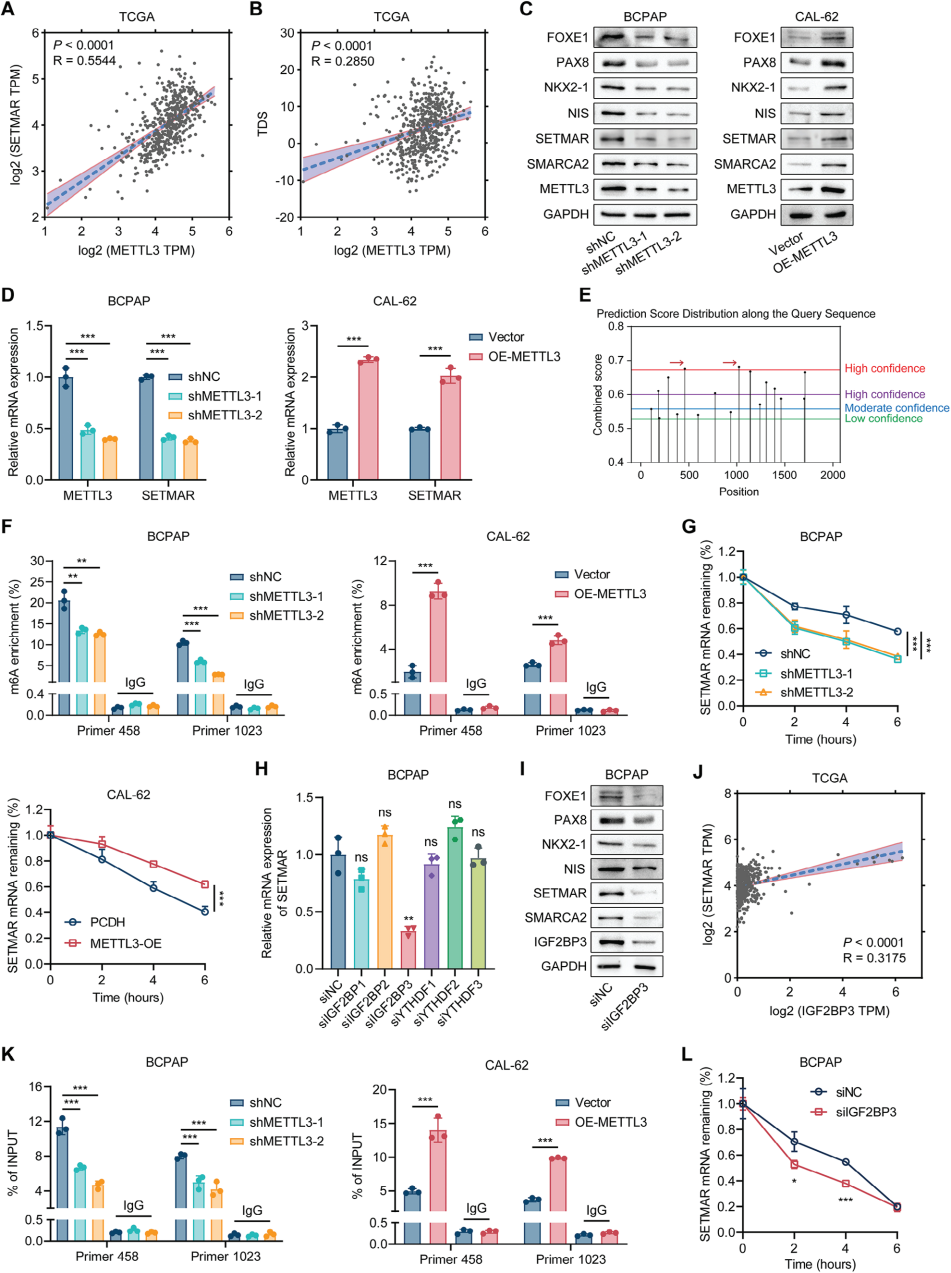
METTL3‐mediated m6A modification stabilizes SETMAR mRNA in an IGF2BP3‐dependent manner. A) Pearson's correlation analysis of METTL3 expression and SETMAR expression in the TCGA database. B) Pearson's correlation analysis of METTL3 expression and TDSs in the TCGA database. C) Western blotting was conducted to evaluate the effects of METTL3 overexpression or knockdown on the protein expression of SETMAR, SMARCA2, and thyroid differentiation markers in thyroid cancer cells. D) RT‒qPCR was performed to assess the effects of METTL3 overexpression or knockdown on the mRNA expression of SETMAR in thyroid cancer cells. E) SRAMP was used to predict the m6A modification site in the SETMAR mRNA. The highly confident m6A modification site is indicated by the red arrow. F) MeRIP‐qPCR verified the presence of METTL3‐mediated m6A modification at the m6A modification prediction site. G) Actinomycin D was administered to both METTL3‐knockdown and METTL3‐overexpressing thyroid cancer cells. The impact of METTL3 expression on SETMAR mRNA stability was assessed using RT‒qPCR. H,I) After silencing the m6A “readers” with distinct siRNAs, RT‒qPCR was used to measure SETMAR expression (H), and Western blotting was used to measure the expression of SETMAR and thyroid differentiation markers (I). J) Pearson's correlation analysis of METTL3 expression and SETMAR expression in the TCGA database. K) RIP‐qPCR confirmed the binding of IGF2BP3 to the m6A modification of SETMAR mRNA. L) Actinomycin D was administered to IGF2BP3‐knockdown BCPAP cells. The impact of IGF2BP3 expression on SETMAR mRNA stability was measured using qPCR. Data are shown as the mean ± SD of three replicates in (D, F, G, H, K, and L). *p* values were determined using a two‐tailed unpaired Student's *t*‐test (^*^ *p* < 0.05, ^**^ *p* < 0.01, ^***^ *p* < 0.001).

METTL3‐mediated m6A modification can affect the mRNA expression profile of tumor cells and widely regulate the biological functions of tumors. By analyzing the TCGA database, we observed a strong correlation between the expression of METTL3 and the expression of SETMAR (**Figure** **7**A), as well as a positive correlation between the expression of METTL3 and the TDS (Figure 7B). The immunohistochemistry results from our PTC tissue cohort also confirmed that METTL3 was positively correlated with the expression of SETMAR, SMARCA2, and NIS (Figure S8A,B, Supporting Information). Furthermore, when examining the expression of METTL3 at the single‐cell level, we observed significantly lower METTL3 levels in tumors, particularly ATC, than in normal thyroid cells(Figure S9A,B, Supporting Information). In light of these results, we hypothesized that METTL3‐mediated m6A modifications are involved in controlling SETMAR expression and thus enhancing thyroid carcinoma differentiation.

We utilized two distinct shRNAs to silence METTL3 in BCPAP cells and overexpressed METTL3 in CAL‐62 cells. Our findings demonstrated that knockdown of METTL3 suppressed the protein and mRNA expression of SETMAR, whereas overexpression of METTL3 substantially increased the protein and mRNA expression of SETMAR (Figure 7C,D). Furthermore, we also found that METTL3 knockdown or overexpression affected the expression of SMARCA2 and thyroid differentiation markers (Figure 7C). The SRAMP (http://www.cuilab.cn/sramp) was used to predict the m6A modification sites of SETMAR mRNA, resulting in the identification of two highly confident m6A modification sites (Figure 7E). Subsequently, we designed qPCR primers for these two sites and performed MeRIP‐qPCR experiments. The results confirmed the occurrence of METTL3‐mediated m6A modification in the region where these two sites are located (Figure 6F). To investigate the impact of METTL3 expression on SETMAR mRNA stability, we added actinomycin D into both METTL3‐knockdown and METTL3 overexpressing cell lines. According to our findings, SETMAR stability decreased with METTL3 knockdown but increased with METTL3 overexpression (Figure 7G). To investigate which m6A sites play essential roles in regulating the mRNA stability of SETMAR, we designed three mutants based on the 2 predicted m6A sites (Figure S9C, Supporting Information). The results of the luciferase report assay showed that the relative luciferase activity decreased obviously in WT after silencing METTL3. However, the luciferase activity of the control group decreased in Mut1, Mut2, and Mut1+2 compared with WT, and the luciferase activity had no significant change when we silenced METTL3, indicating that both the two m6A sites are important for regulating mRNA stability of SETMAR (Figure S9D, Supporting Information). To further confirm that METTL3 regulates thyroid cancer differentiation through the SETMAR‐SMARCA2‐TTF axis, we knocked down SETMAR in METTL3 overexpressed ATC cells and found that the up‐regulated SMARCA2 and thyroid differentiation markers caused by METTL3 overexpression can be rescued by SETMAR silencing (Figure S9E, Supporting Information).

To identify m6A “readers” that specifically bind to the m6A modification on SETMAR mRNA, we used siRNA targeting distinct m6A “readers” to individually suppress their expression and assess the resulting changes in SETMAR expression (Figure S9F, Supporting Information). These m6A “readers” influence mRNA stability by recognizing m6A modifications.^[^ ^26^ ^]^ Notably, suppression of IGF2BP3 led to a significant reduction in SETMAR expression (Figure 7H), suggesting that IGF2BP3 may serve as a m6A “reader” for SETMAR. We further confirmed that downregulating IGF2BP3 expression also led to a decrease in the expression of SMARCA2 and indicators related to thyroid differentiation (Figure 7I). Additionally, our analysis of the TCGA database revealed a positive correlation between the expression of IGF2BP3 and that of SETMAR as well as between the expression of IGF2BP3 and that of SMARCA2 (Figure 7J; Figure S9G, Supporting Information). We also conducted RIP‐qPCR to confirm the binding of IGF2BP3 to the two m6A modification sites on SETMAR mRNA (Figure 7K). Similarly, we added actinomycin D to IGF2BP3‐knockdown cells and found that IGF2BP3 knockdown significantly reduced the stability of SETMAR (Figure 7L), while knocking down IGF2BP3 in METTL3 overexpressing cells reversed the METTL3 overexpression‐induced increase in SETMAR expression and SETMAR mRNA stability (Figure S9H,I, Supporting Information).

In conclusion, the expression of SETMAR in thyroid cancer is stabilized by METTL3‐mediated m6A modification in an IGF2BP3‐dependent manner, which could explain its dysregulation during dedifferentiation of thyroid cancer.

### 2.7 SETMAR Reinforces the Redifferentiation Effects of MAPK Inhibition

It has been reported that the deletion of SWI/SNF complex subunits can increase the resistance of thyroid cancer to the redifferentiation effect of MAPK inhibition.^[^ ^21^ ^]^ We wanted to verify whether the regulatory effect of SETMAR on the expression of SWI/SNF complex subunits can affect the sensitivity of thyroid cancer cells to MAPK inhibitor‐based redifferentiation therapies.

**Figure 8:**
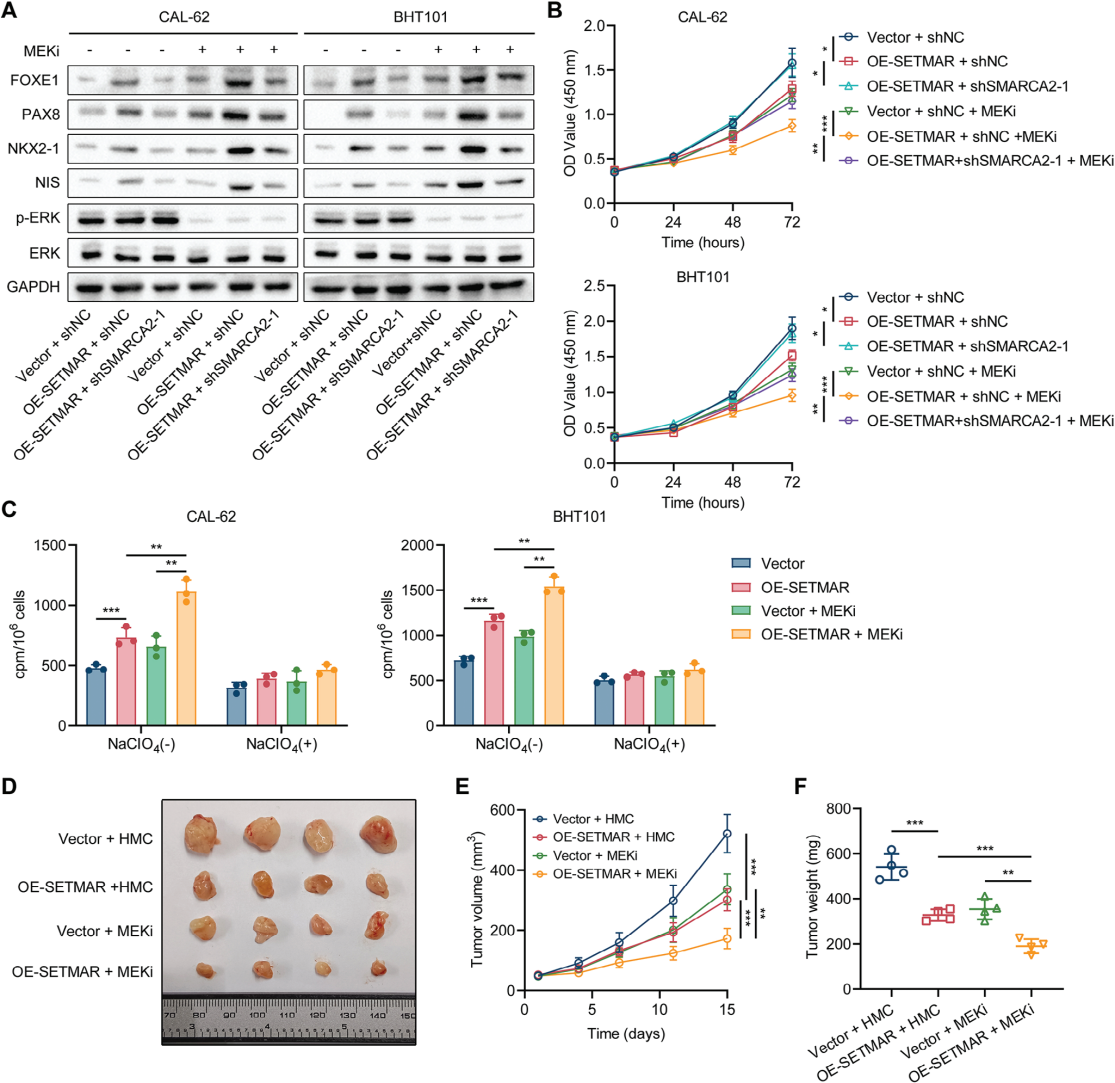
SETMAR reinforces the redifferentiation effects of MAPK inhibition. A) Western blotting was used to measure the expression of thyroid differentiation markers in ATC cells with SETMAR overexpression or SETMAR overexpression accompanied by SMARCA2 knockdown with or without selumetinib treatment (1 µM) for 24 h. B) A CCK‐8 assay was used to determine the proliferation of ATC cells with SETMAR overexpression or SETMAR overexpression accompanied by SMARCA2 knockdown with or without selumetinib treatment (1 µM). C) Radioactive iodine uptake was measured in ATC cells with or without SETMAR overexpression treated with or without selumetinib (1 µM) for 24 h. D) Subcutaneous nude mouse xenografts formed by SETMAR‐overexpressing or control CAL‐62 cells were photographed after treatment with or without selumetinib. When the average tumor volume reached 50 cm^3^, selumetinib was intragastrically administered at a dose of 10 mg k^−1^g once daily. *n* = 4 animals per group. Subcutaneous xenografts were dissected on Day 15 and analyzed. E) Growth curves of the subcutaneous xenografts in each group. F) Tumor weights of each group. Data are shown as the mean ± SD of three replicates in (B, C) and four replicates in (E, F). *p* values were determined using two‐tailed unpaired Student's *t*‐test (^*^ *p* < 0.05, ^**^ *p* < 0.01, ^***^ *p* < 0.001).

Our study revealed that overexpression of SETMAR or SMARCA2 in ATC cells significantly enhanced the redifferentiation effects of MAPK inhibitor, as indicated by increased thyroid differentiation marker expression and decreased proliferation (**Figure** **8**A,B; Figure S10A,B, Supporting Information). However, SETMAR overexpression accompanied by SMARCA2 knockdown strongly impaired the SETMAR‐enhanced redifferentiation effect of MAPK inhibitor (Figure 8A,B). Moreover, the overexpression of SETMAR enhanced the increase in radioactive iodine uptake induced by MAPK inhibitors (Figure 8C). We also found that compared with wild‐type SETMAR, overexpression of methyltransferase‐deficient SETMAR did not enhance the redifferentiation‐inducing effect of MAPK (Figure S10C,D, Supporting Information).

Finally, we assessed the impact of SETMAR on MAPK inhibitor‐induced redifferentiation in vivo. The results showed that SETMAR overexpression significantly enhanced the suppressive effect of MAPK inhibitors on tumor proliferation (Figure 8D–F). Immunohistochemical staining confirmed that SETMAR promoted the MAPK inhibitor‐induced increased expression of thyroid differentiation markers in vivo (Figure S10E, Supporting Information).

### 2.8 The METTL3‐14‐WTAP Activator Promotes Thyroid Cancer Redifferentiation via the SETMAR‐SMARCA2‐TTF Axis

**Figure 9:**
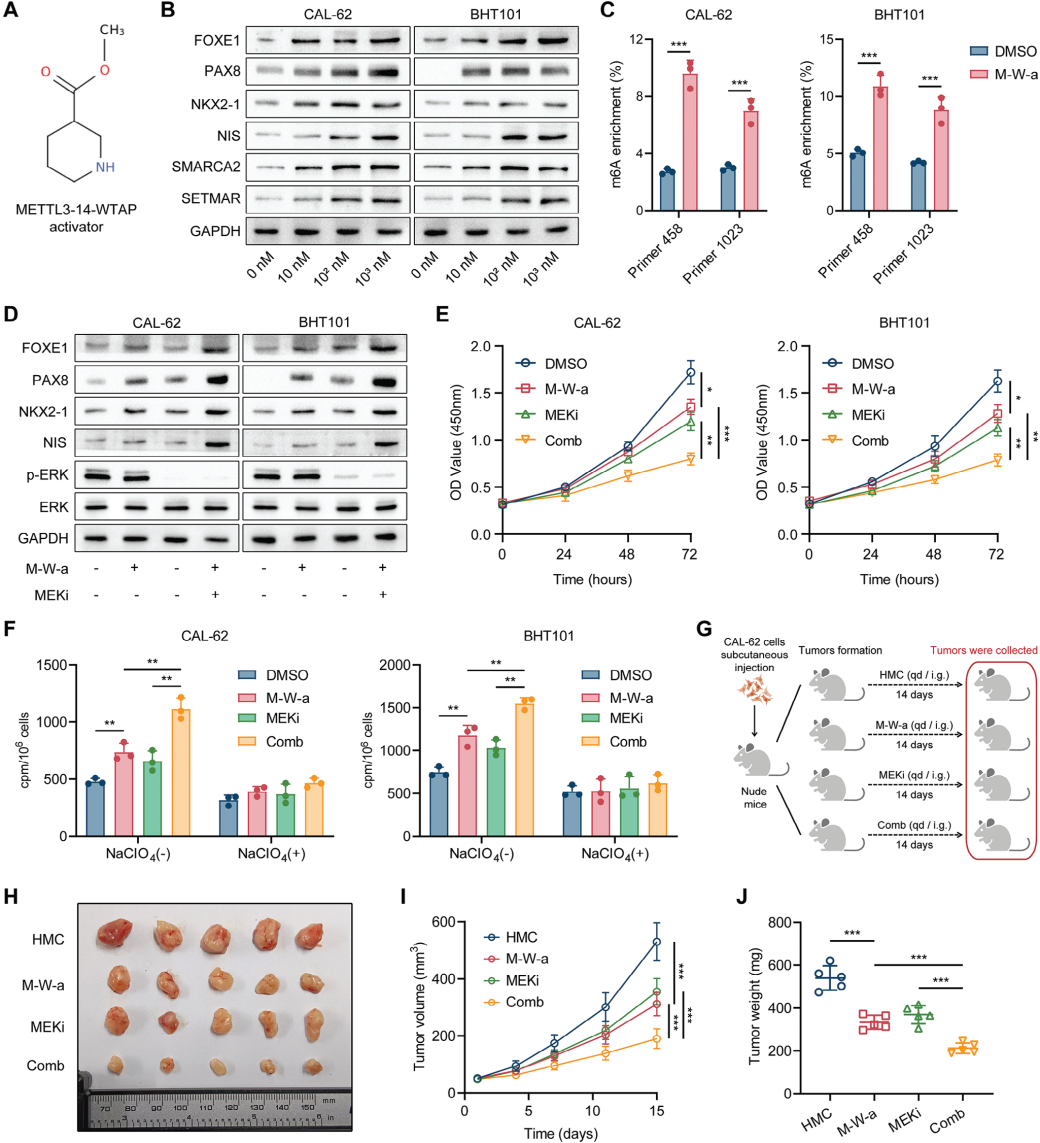
The METTL3‐14‐WTAP activator promotes thyroid cancer redifferentiation via the SETMAR‐SMARCA2‐TTF axis. A) The structure of the METTL3‐14‐WTAP activator (M‐W‐a). B) The effect of treatment with various concentrations of METTL3‐14‐WTAP activator for 24 h on the expression of SETMAR, SMARCA2, and thyroid differentiation markers in ATC cells was investigated by Western blotting. C) MeRIP‐qPCR was used to quantify the abundance of m6A modifications on SETMAR mRNA after treatment with or without the METTL3‐14‐WTAP activator (100 nM) for 24 h. D) Western blotting was used to evaluate the effects of treatment with the METTL3‐14‐WTAP activator (100 nM) and selumetinib (1 µM) alone or in combination for 24 h on the expression of thyroid differentiation markers in ATC cells. E) A CCK‐8 assay was conducted to evaluate the effects of treatment with the METTL3‐14‐WTAP activator (100 nM) and selumetinib (1 µM) alone or in combination on the proliferation of ATC cells. F) A radioactive iodine uptake assay was performed to assess the impact of treatment with the METTL3‐14‐WTAP activator (100 nM) and selumetinib (1 µM), either alone or in combination, for 24  on the ability of ATC cells to take up ^131^I. G) The diagrammatic sketch of constructing subcutaneous xenograft models in nude mice, followed by treatment with the indicated drug. *n* = 5 mice per group. When the average tumor volume reached 50 cm^3^, treatment was performed by the intragastric administration of 10 mg k^−1 ^g^−1^ selumetinib or 10 mg k^−1 ^g^−1^ METTL3‐14‐WTAP activator once a day from Day 1 to Day 14. Subcutaneous xenografts were dissected on Day 15. H) Subcutaneous xenografts that were derived from CAL‐62 cells that were treated with the METTL3‐14‐WTAP activator, selumetinib or their combination were harvested from nude mice and photographed. I) Growth curves of subcutaneous xenografts in each group. J) Tumor weights of xenografts in each group. Data are shown as the mean ± SD of three replicates in (C, E, and F) and five replicates in (I, J). *p* values were determined using a two‐tailed unpaired Student's *t*‐test (^*^ *p* < 0.05, ^**^ *p* < 0.01, ^***^ *p* < 0.001).

Dysregulation of METTL3 expression has been associated with various cancers and pharmacological modulation of RNA methylation using small molecule modulators (inhibitors and/or activators) has great therapeutic potential for supporting cancer therapy.^[^ ^27^ ^]^ Therefore, we studied the efficacy of a previously reported METTL3‐14‐WTAP activator (**Figure** **9**A)^[^ ^28^ ^]^ in the treatment of thyroid cancer, thus providing an innovative approach for the redifferentiation treatment of thyroid cancer.

First, we synthesized levorotatory and dextral drug monomers of the METTL3‐14‐WTAP activator and examined their ability to induce thyroid cancer redifferentiation. We observed that the dextral METTL3‐14‐WTAP activator has better activity in promoting redifferentiation of thyroid cancer, as indicated by a more pronounced promotion effect on the mRNA expression of SETMAR, SMARCA2, and thyroid differentiation markers in ATC cells (Figure S11A, Supporting Information), and it more strongly inhibited the viability of ATC cells (Figure S11B, Supporting Information). Therefore, we chose the dextral monomer for follow‐up experiments. We found that dextrorotatory activator dose‐dependently enhanced the protein expression of SETMAR, SMARCA2, and thyroid differentiation markers in ATC cells (Figure 9B). Following METTL3‐14‐WTAP activator treatment, there was a significant increase in the m6A modification level of SETMAR mRNA, as determined by MeRIP‐qPCR (Figure 9C). Moreover, treatment with the METTL3‐14‐WTAP activator decreased the migration and invasion of ATC cells (Figure S11C, Supporting Information). Treatment with the METTL3‐14‐WTAP activator also reinforced the redifferentiation effect of the MAPK inhibitor, as indicated by the increased expression of thyroid differentiation markers (Figure 9D), stronger inhibition of proliferation (Figure 9E), and increased radioactive iodine uptake (Figure 9F). Importantly, the effects of the METTL3‐14‐WTAP activator on promoting thyroid differentiation were reversed in ATC cells when SETMAR or SMARCA2 was knocked down, confirming the on‐target effects of the METTL3‐14‐WTAP activator (Figure S11D, Supporting Information).

Finally, we evaluated the effectiveness of the METTL3‐14‐WTAP activator in treating thyroid cancer in vivo. We treated nude mice which bear subcutaneous xenograft tumors formed by CAL‐62 cells with single agents or a combination of METTL3‐14‐WTAP activators and MAPK inhibitors (Figure 9G). Treatment with the METTL3‐14‐WTAP activator effectively suppressed tumor growth in vivo (Figure 9H–J) and stimulated the differentiation of thyroid cancer through the METTL3‐SETMAR‐SMARCA2‐TTF axis, which was confirmed by increased immunohistochemical staining for SETMAR, SMARCA2 and thyroid differentiation markers in tumors after treatment (Figure S12A, Supporting Information). Moreover, combination treatment with MAPK inhibitors further augmented these effects (Figure S12A, Supporting Information). In addition, METTL3‐14 WTAP activator did not influence the body weight of the mice (Figure S12B, Supporting Information), and H&E staining of mouse lungs, livers, hearts, and kidneys revealed no obvious drug toxicity (Figure S12C, Supporting Information), suggesting that the METTL3‐14‐WTAP activator has good pharmacological safety. These data suggest that the METTL3‐14‐WTAP activator can suppress thyroid cancer progression and promote differentiation through the pharmacological activation of SETMAR. Therefore, targeting SETMAR may constitute a new therapeutic strategy for patients diagnosed with progressive thyroid carcinoma.

## 3 Discussion

**Figure 10:**
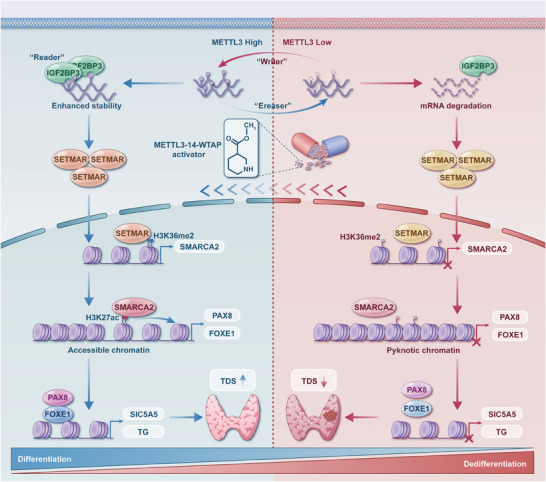
Schematic model of METTL3‐SETMAR‐SMARCA2‐TTF axis‐dependent regulation of thyroid cancer differentiation. In well‐differentiated thyroid cancer, METTL3‐mediated m6A modification on SETMAR mRNA increases its stability in an IGF2BP3‐dependent manner. SETMAR methylates dimethylated H3K36 in the SMARCA2 promoter region to promote SMARCA2 transcription. SMARCA2 can bind to enhancers of the thyroid differentiation transcription factors (TTFs) PAX8, and FOXE1 to promote their expression by enhancing chromatin accessibility. Dysregulation of the expression of key genes in this axis, however, leads to the dedifferentiation of thyroid cancer. The METTL3‐14‐WTAP activator can restore SETMAR expression and promote differentiation of thyroid cancer, thus providing therapeutic benefits. The schematic figure was drawn by Figdraw (www.figdraw.com).

In this study, we identified SETMAR as a differentiation‐related histone methylation modifier in thyroid cancer, and we found that its mRNA was stabilized by METTL3‐mediated m6A modification in an IGF2BP3‐dependent manner. The underlying mechanism involves the ability of SETAMR to methylate dimethylated H3K4 in the SMARCA2 promoter region, thereby promoting its transcription. SMARCA2 anchors to the enhancers of PAX8 and FOXE1, increasing chromatin accessibility and promoting the transcription of these genes (**Figure** **10**). Finally, we employed the METTL3‐14‐WTAP activator to facilitate thyroid cancer differentiation by increasing SETMAR expression. Based on the current difficulties in the treatment of refractory thyroid cancer, we conducted a comprehensive investigation into the upstream and downstream networks of SETMAR that regulate thyroid cell differentiation. Our study revealed the key epigenetic mechanism by which SETMAR regulates the differentiation of thyroid cancer cells. Importantly, we propose a novel model for understanding the regulation of tumor differentiation in thyroid cancer, and this model involves RNA m6A methylation, histone methylation, and chromatin remodeling. This model offers a new method for treating advanced thyroid cancer by promoting differentiation and provides valuable insights for future research in this field. In our research, the absence of pairwise comparison outcomes among classic PTCs, follicular PTCs, and tall‐cell PTCs stemmed from the scarcity of the high‐cell subtype in our clinical dataset. Nevertheless, it's crucial to note that this limitation does not compromise the integrity of our primary finding.

Cancer progression is a dynamic process of molecular events that lead to genetic instability and abnormal phenotypes.^[^ ^29^ ^]^ A distinct feature of cancer progression is the dedifferentiation of cancer cells. This process leads to increased proliferation of tumor cells, as well as their ability to metastasize and resist to conventional treatments, such as radiotherapy, chemotherapy, and targeted therapy.^[^ ^30^, ^31^ ^]^ The development of tumor redifferentiation therapy is essential for improving cancer treatment strategies. Redifferentiation therapy has made remarkable strides in treating a variety of malignancies, including thyroid cancer. In addition, promyelocytic leukemia (PML) and retinoic acid receptor α gene (RARα) fuse to form the PML‐RARα fusion protein, which prevents myeloid cell differentiation at the promyelocyte stage and causes acute promyelocytic leukemia (APL).^[^ ^32^ ^]^ Treatment with all‐trans retinoic acid and arsenic trioxide has significantly transformed the management of this disease.^[^ ^33^ ^]^ On the other hand, mutations in the IDH1 and IDH2, genes produce the oncometabolite 2‐hydroxyglutarate, which impairs differentiation and leads to the development of acute myeloid leukemia.^[^ ^34^, ^35^ ^]^ Small molecule inhibitors that target IDH1 and IDH2 can induce myeloid differentiation and offer therapeutic benefits to these patients.^[^ ^36^ ^]^ Moreover, selective cytokinin‐activated protein kinase (MAPK) pathway inhibitors have been found to enhance sodium iodide symporter synthesis and facilitate iodine uptake in thyroid cancer. In patients with radioiodine‐resistant thyroid cancer, the administration of selumetinib has been shown to exert a notable clinical impact on augmenting iodine uptake and retention.^[^ ^37^, ^38^ ^]^ Therefore, differentiation treatment based on targeted therapy is expected to become one of the fundamental pillars of future tumor treatment.

Our study revealed that histone methylation plays a crucial role in the regulation of thyroid cancer differentiation. Numerous enzymes that are involved in histone methylation, such as SETMAR, have been found to be associated with the differentiation of thyroid cancer. SETMAR, also known as METnase, is a fusion protein between a SET domain protein methylase and the transposase Hsmar1.^[^ ^15^ ^]^ Previous studies have shown that SETMAR plays an important role in different cellular processes, such as nonhomologous end joining (NHEJ), the integration of transfected plasmids and lentiviruses, chromosome decatenation, and the regulation of gene expression.^[^ ^14^ ^]^ However, how SETMAR functions in thyroid cancer has not been determined. Here, we first discovered that SETMAR regulates the transcription of numerous SWI/SNF complex subunits either directly or indirectly. This enables SETMAR to modulate SWI/SNF complex‐mediated chromatin remodeling, which in turn allows it to effectively control developmental processes and determine lineage specification. This crucial characteristic of SETMAR also enables it to control the differentiation of thyroid cancer, which has a significant impact on cancer progression. However, we did not thoroughly examine how SETMAR controls the expression of SWI/SNF subunits other than SMARCA2, especially its indirect downstream targets.

SWI/SNF subunits are commonly found to be mutated in human cancers.^[^ ^39^ ^]^ These mutations can result in various changes in cancer characteristics, such as abnormal cell proliferation, lineage differentiation, and metabolic alterations.^[^ ^40^, ^41^ ^]^ A previous study demonstrated that the SWI/SNF complex is critical for maintaining differentiation status in thyroid cancer, and its mutations, including those in Arid1a, Arid2, or Smarcb1, lead to resistance to radioactive iodine and MAPK inhibitor‐based differentiation therapy.^[^ ^21^ ^]^ However, the role of SMARCA2, which is the core component of the SWI/SNF complex responsible for providing energy for chromatin remodeling, has not been elucidated in the context of thyroid cancer. Our study revealed that SMARCA2 could regulate the chromatin accessibility of key thyroid lineage transcription factors through chromatin remodeling, thus regulating thyroid cancer differentiation. Notably, SMARCA4 and SMARCA2 are two important mutually exclusive catalytic subunits in the SWI/SNF complex, and they have a high degree of homology.^[^ ^42^ ^]^ In addition, the paralog dependency model has been employed to elucidate the SWI/SNF complex, where SMARCA2 and SMARCA4 can compensate for the absence of one another.^[^ ^24^ ^]^ As a result, understanding how SMARCA4 functions in thyroid cancer may be of significant scientific benefit.

M6A methylation is a common mRNA modification that regulates target genes through “three components”, namely, the “writer”, which mediates methylation, the “eraser”, which mediates demethylation, and the “reader”, which is responsible for recognizing the methylation reaction.^[^ ^43^ ^]^ METTL3 is a “writer”. Previous studies of METTL3 in thyroid cancer have shown that reduced METTL3 expression is associated with a reduced response to immune checkpoint blockade. Overexpression of METTL3 in cancer cells can destabilize CD70 mRNA in a YTHDF2‐dependent manner, thereby improving the efficacy of anti‐PD‐1 therapy in thyroid cancer.^[^ ^44^ ^]^ Moreover, METTL3 inhibited the progression of papillary thyroid cancer through m6A/c‐Rel/IL‐8‐mediated neutrophil infiltration.^[^ ^45^ ^]^ Nevertheless, no studies have shed light on the function or mechanism of METTL3‐mediated m6A methylation in thyroid carcinoma differentiation. This work is the first to demonstrate the important function of the METTL3‐regulated epigenetic network in controlling the differentiation of thyroid cancer. Additionally, by focusing on METTL3, we discovered a novel method for treating thyroid cancer differentiation, providing new therapeutic options for this disease.

## 4 Conclusion

In conclusion, we demonstrated that the control of thyroid differentiation‐related transcription factors via the METTL3‐SETMAR‐SMARCA2 axis contributes to the differentiation of thyroid cancer. Targeting this process by targeting important related genes may be a useful therapeutic intervention strategy. For patients with advanced thyroid cancer, a combination regimen consisting of radioactive iodine therapy, a METTL3‐14‐WTAP small molecule activator, and MAPK inhibitor‐based redifferentiation therapy may be a potential therapeutic approach.

## 5 Experimental Section

### Cell Lines and Cell Culture

The CAL‐62, C643, BCPAP, and ACT‐1 cell lines were purchased from the American Type Culture Collection (USA). The K1, TPC1, KTC‐1, and BHT101 cell lines were purchased from Guangzhou Cellcook Biotech (China). The Nthy‐ori 3–1 and IHH4 cell lines were purchased from the Chinese Academy of Science (China). All cell lines have been authenticated by STR analysis. All the cell lines were cultured in RPMI‐1640 medium or DMEM medium supplemented with 10% fetal bovine serum and maintained at 37 °C in a humidified environment with 5% CO_2_.

### Clinical Data and Tissue Samples

This study used tumor tissue sections from 124 patients whose medical records were complete. These patients were diagnosed with PTC, PDTC, or ATC at Tianjin Medical University Cancer Institute and Hospital. Matched fresh PTC tissues and adjacent normal thyroid tissues were obtained from 26 patients. Total RNA was isolated from 16 paired tissue samples, and total protein was isolated from 10 paired tissue samples. The Ethics Committee of Tianjin Medical University Cancer Hospital approved this study (Approval ID: Ek2021149). Informed consent was acquired to conduct the experiments using human tissues.

### Total RNA Extraction, Reverse Transcription‐Quantitative PCR(RT‐qPCR), and mRNA Stability Assays

Total RNA was extracted from thyroid cancer cells and fresh tissues using an RNA extraction solution (G3013; Servicebio, China). The mRNA was reverse transcribed into cDNA using a HiScript II 1st Strand cDNA Synthesis Kit (R212‐01, Vazyme, China). Quantitative real‐time PCR was performed with HiScript II Q RT SuperMix for qPCR (R223‐01, Vazyme, China) and specific primers according to the instructions. Relative RNA expression was measured using the 2^−ΔΔCt^ method and β‐actin was used as an internal control for normalization. The sequences of the primers used are listed in Additional file 1.1. To measure the stability of the mRNA in different cells, 5 mg mL^−1^ actinomycin D was added. After treatment for the specified times, the cells were collected, and total RNA was extracted for RT‐qPCR to quantify mRNA stability.

### Western Blotting

Cells or harvested tissues were lysed with RIPA lysis buffer (R0020, Solarbio, China), and the protein concentrations were measured using the BCA Protein Assay Kit (PC0020, Solarbio, China). The samples were then mixed with protein sample loading buffer (LT 101, Epizyme, China) and boiled to prepare them for SDS‐PAGE. The proteins in the gels were transferred to PVDF membranes (WJ002, Epizyme, China) using a standard Bio‐Rad wet membrane transfer device. Then, the PVDF membranes were blocked with 5% skim milk (D8304, Solarbio, China) and incubated with primary antibodies at 4 °C overnight. The next day, after the PVDF membranes had been incubated with the secondary antibodies, they were subjected to chemiluminescence detection using an Omni‐ECLFemto Light Chemiluminescence Kit (SQ201, Solarbio, China).

### Cell Transfection and Lentivirus Infection

Small interfering RNAs (siRNAs) or plasmids were transfected into cells using Lipofectamine 3000 (L3000075, Thermo Fisher Scientific, USA), and siRNAs were synthesized by Hanbio Biotechnology (China). Lentiviruses for knockdown and overexpression (shSETMAR, shSMARCA2, shMETTL3, OE‐SETMAR, and OE‐METTL3) were purchased from Hanbio Biotechnology (China). The lentivirus‐infected cancer cells were selected with 1 µg mL^−1^ puromycin. For SMARCA2 overexpression, we used the CRISPR transcriptional activation system to construct endogenous SMARCA2 overexpressed ATC cell lines. In brief, ATC cells were infected with a lentivirus generated by transfecting 293T cells with the vector lentimphv2 and selected with 500 µg mL^−1^ hygromycin B. The cells were then transduced with lentiSAMv2 carrying a sgRNA that was specific for the SMARCA2 gene and then selected with blasticidin S (5 µg mL^−1^). The sequences of the siRNAs, shRNAs, and sgRNAs that were used are listed in Additional file 1.2.

### In Vitro ^131^I Uptake Assay

A total of 5 × 10^5^ thyroid cancer cells were seeded in triplicate in 12‐well plates. The cells were incubated in a serum‐free medium containing 1 µCi Na^131^I and 10 µM NaI^−1^ for 30 min at 37 °C. In the control group, the cells were pretreated with the competitive NIS inhibitor NaClO_4_ (300 µM) for 30 min before Na^131^I treatment, so the nonspecific uptake of radioactive iodine could be measured. The cells were then washed with ice‐cold PBS and lysed in 0.5 mL of 0.3 M NaOH. The radioactivity of each sample was measured with a Perkin Elmar 2470 gamma counter.

### RNA‐Seq Analysis

SETMAR‐overexpressing or control CAL‐62 cells were lysed using an RNA extraction solution (G3013; Servicebio, China) and the mRNA was extracted according to the manufacturer's protocol. The global gene expression profiles were determined by mRNA sequencing, which was performed at Majorbio Bio‐Pharma Technology (China). Bioinformatic analyses were performed as previously described.^[^ ^46^ ^]^ The raw RNA‐Seq data were uploaded to the Sequence Read Archive (SRA) under accession number PRJNA1067444.

### CUT&Tag Assay

The CUT&Tag assay was conducted using the Hyperactive Universal CUT&Tag Assay Kit for Illumina (TD903, Vazyme, China). In brief, SETMAR‐overexpressing or control CAL‐62 cells were harvested. Con A magnetic beads were used for cell capture, while 5% Digitonin was utilized to permeabilize the cell membrane. The DNA sequences that bound to the target protein were specifically cleaved by combining primary antibodies, secondary antibodies, and the Protein A‐Transposome. Subsequently, PCR was employed to assemble a second‐generation sequencing library, enabling the acquisition of a high‐resolution map of the genes that bound to the target protein via next‐generation sequencing. Libraries were sequenced on a NovaSeq PE150 by Novogene (Beijing, China). CUT&Tag data were aligned to the GRCh37/hg19 human reference genome using SOAPaligner/SOAP2 (Short Oligonucleotide Analysis Package). A maximum of two mismatches were allowed in a pair. Reads that mapped only once at a specific location were used for peak calling. The peaks from CUT&Tag were identified using MACS software, specifically version MACS‐1.4.2. Big‐wig files were generated using MACS‐1.4.2. CUT&Tag tracks were visualized using IGVtools (version 2.11.3). The raw CUT&Tag data were uploaded to the Sequence Read Archive (SRA) under accession number PRJNA1067517.

### Chromatin Immunoprecipitation (ChIP)

For each ChIP sample, 1 × 10^6^ cells were crosslinked with 1% formaldehyde, and cross‐linked chromatin was sonicated to obtain DNA fragments between 200 and 500 bp for immunoprecipitation. The protein/DNA complexes were then separated and purified. Purified DNA was used for qPCR analysis or to generate a second‐generation sequencing library, which was subsequently sequenced on a NovaSeq PE150 by Novogene (Beijing, China). The ChIP‐seq data analysis was conducted as previously described.^[^ ^33^ ^]^ The raw ChIP‐seq data were uploaded to the Sequence Read Archive (SRA) under accession number PRJNA1067866. The sequences of the primers that were used for ChIP‐qPCR are listed in Additional file 1.3.

### ATAC‐seq

The ATAC‐seq assay was performed using the Hyperactive ATAC‐Seq Library Prep Kit for Illumina (TD711, Vazyme, China). In brief, 1 × 10^4^ SMARCA2‐overexpressing or control CAL‐62 cells were washed with PBS and resuspended in lysis buffer. The cell lysates are subsequently centrifuged to pellet the DNA. The pelleted DNA was subjected to transposition with Tn5 transposase by resuspending it in a transposition reaction buffer. DNA was purified and used to construct ATAC libraries. The libraries were sequenced on NovaSeq PE150 by Novogene (Beijing, China). Motif signatures were obtained using Homer v4.5 (http://homer.ucsd.edu). Tornado plots were generated using deepTools (92) v3.3.0 software. The standardized bigwigs were processed through the computeMatrix and plotHeatmap functions. The average signal was sampled over a 25 bp window, and the flanking regions of 5 kb were defined. The sequencing data were uploaded to the Sequence Read Archive (SRA) under accession number PRJNA1067869.

### Chromosome Conformation Capture (3C) Assay

1 × 10^7^ cells were cross‐linked with 1% formaldehyde for 10 min at room temperature and quenched with 125 mM glycine. Nuclei were lysed using pre‐cold 3C lysis buffer (10 mM NaCl, 0.2% NP‐40, 5 mM MgCl_2_, 0.1 mM EGTA, 10 mM Tris‐HCl, pH 7.5, 1 mM PMSF). Cells were digested with 200 U Dpn II (NEB) at 37 °C, 900 rpm overnight. The digested products were ligated with 400 U T4 DNA ligase (Takara) at 16 °C overnight. The digestion and ligase efficiency were determined using 1% agarose. The ligated DNA was de‐crosslinked with proteinase K at 65 °C overnight. The DNA was extracted using a DNA extraction buffer (Solarbio) and quantified by Nanodrop. The 3C products were amplified using PCR with a pair of primers shown in Additional file 1.4.

### MeRIP‐qPCR

Total RNA was isolated from cancer cells, and the volume was adjusted to 18 µL with RNase‐free water. The amount of total RNA in each sample was 5 µg. Then, 2 µL of 10 × RNA Fragmentation Buffer (100 mM Tris‐HCl, 100 mM ZnCl_2_ in RNase‐free water) was added and incubated at 70 °C for 5–6 min to fragment the total RNA into ≈200 nt fragments. Then, 2 µL of 0.5 M EDTA was added to terminate the reaction. 30 µL of A/G magnetic beads (#P2029, Beyotime, China) were washed twice with IP buffer (150 mM NaCl, 10 mM Tris‐HCl, pH 7.5, 0.1% IGEPAL CA‐630). The beads were then resuspended in 500 µL of IP Buffer containing 1 µg of anti‐m6A antibody (202 003, Synaptic Systems, Germany) or normal rabbit IgG (#2729, Cell Signaling Technology). The mixture was rotated overnight at 4 °C. The antibody‐bead complexes were then resuspended in 500 µL of IP mixture containing fragmented RNA and 5 µL of RNase inhibitor (K1046, APExBIO, USA) and incubated for 3 h at 4 °C. The RNA reaction mixture was then washed with low/high salt wash buffer. m6A‐enriched RNA was isolated with 16 µL of nuclease‐free water according to the instructions of the RNeasy Mini Kit (QIAGEN) and subjected to RT‐qPCR. The primers that were used for MeRIP‐qPCR are listed in Additional file 1.5.

### Drugs, and Antibodies

All the drugs and antibodies that were used in this study are listed in Additional file 1.6.

### Animal Studies

All animal experiments were approved by the Ethics Committee of the Tianjin Medical University Cancer Institute and Hospital (Approved No.: NSFC‐AE‐2021199). All mice were purchased from SPF Biotechnology (Beijing, China). 1 × 10^6^ CAL‐62 cells were subcutaneously injected into 6‐week‐old female BALB/c nude mice to establish tumor xenograft models. Tumor growth was measured every 2–3 days after the mean tumor size reached 50 mm^3^. The tumor volumes were calculated as follows: Tumor volume = length × width^2^/2

### Statistical Analysis

The experimental data were analyzed using the GraphPad Prism 9.0 software. All the in vitro results were representative of at least three independent experiments and the results were presented as the mean ± SD or median with range. Paired PTC and corresponding normal thyroid samples were analyzed using paired *t*‐tests. Correlations between different genes were analyzed using the Pearson correlation test. Kaplan–Meier analysis was used to evaluate survival curves, and the differences in the survival probabilities were assessed using the log‐rank test. Differential analysis between 2 groups was conducted with the two‐tailed unpaired Student's *t*‐test (^***^ *p* < 0.001, ^**^ *p* < 0.01, ^*^ *p* < 0.05).

Detailed information about the other experiments including Thyroid Differentiation Score (TDS) calculation, analysis of single‐cell datasets, immunohistochemistry (IHC), cell viability assay, Transwell migration, and invasion assay, and RIP‐qPCR, is included in Additional file 2.

## Conflict of Interest

The authors declare no conflict of interest.

## Supporting information

Supporting Information

## Data Availability

The data that support the findings of this study are available from the corresponding author upon reasonable request.
