# Supplementary material for: SETMAR Facilitates the Differentiation of Thyroid Cancer by Regulating SMARCA2‐Mediated Chromatin Remodeling
Source: Adv Sci (Weinh). 2024 Jun 20;11(32):2401712. doi: 10.1002/advs.202401712 (PMC11348079; doi:10.1002/advs.202401712)
Supplement: Supplementary file 1 — Supporting Information [file ADVS-11-2401712-s001.docx]

SETMAR facilitates the differentiation of thyroid cancer by regulating SMARCA2-mediated chromatin remodeling

Wei Zhang^1, 2, 3, 4#^, Xianhui Ruan^2#^, Yue Huang^2^, Weiyu Zhang^5^, Guangwei Xu^2^, Jingzhu Zhao^2^, Jie Hao^2, 3, 4^, Nan Qin^6^, Jinjian Liu^7^, Qian Su^8^, Jianfeng Liu^7^, Mei Tao^2^, Yuqi Wang^2^, Songfeng Wei^2*^, Xiangqian Zheng^2*^, Ming Gao^1, 2, 3, 4*^

1 School of Medicine, Nankai University, Tianjin, China.

2 Department of Thyroid and Neck Tumor, Tianjin Medical University Cancer Institute and Hospital, National Clinical Research Center for Cancer, Key Laboratory of Cancer Prevention and Therapy, Tianjin’s Clinical Research Center for Cancer, Tianjin, China

3 Department of Thyroid and Breast Surgery, Tianjin Union Medical Center, Tianjin, China.

4 Tianjin Key Laboratory of General Surgery in Construction, Tianjin Union Medical Center, Tianjin, China

5 Department of Molecular Biology and Genetics, Cornell University, Ithaca, NY, USA

6 School of Pharmacy, Tianjin Medical University, Tianjin Key Laboratory on Technologies Enabling Development Clinical Therapeutics and Diagnostics (Theragnostic), Tianjin, China.

7 Key Laboratory of Radiopharmacokinetics for Innovative Drugs, Chinese Academy of Medical Sciences, Tianjin Key Laboratory of Radiation Medicine and Molecular Nuclear Medicine, Institute of Radiation Medicine, Chinese Academy of Medical Sciences & Peking Union Medical College, Tianjin, P. R. China.

8 Department of Molecular Imaging and Nuclear Medicine, Tianjin Medical University Cancer Institute and Hospital, National Clinical Research Center for Cancer, Tianjin Key Laboratory of Cancer Prevention and Therapy, Tianjin’s Clinical Research Center for China, Tianjin, China

^#^These authors contributed equally to this work.

^*^Corresponding author:

Ming Gao (E-mail: headandneck2008@126.com)

Xiangqian Zheng (E-mail: [xzheng05@tmu.edu](mailto:xzheng05@tmu.edu).cn)

Songfeng Wei (E-mail: [lidapeng0203@163.com](mailto:lidapeng0203@163.com))

School of Medicine, Nankai University

Weijin Road, Nankai District

Tianjin 300060, China

E-mail: headandneck2008@126.com

**Table of contents**

Supplementary Figures and Supplementary Figure legends ------------------------------ 4

Additional File1 ------------------------------------------------------------------------------- 23

Additional File2 ------------------------------------------------------------------------------- 27

**Supplementary Figures and Supplementary Figure legends**


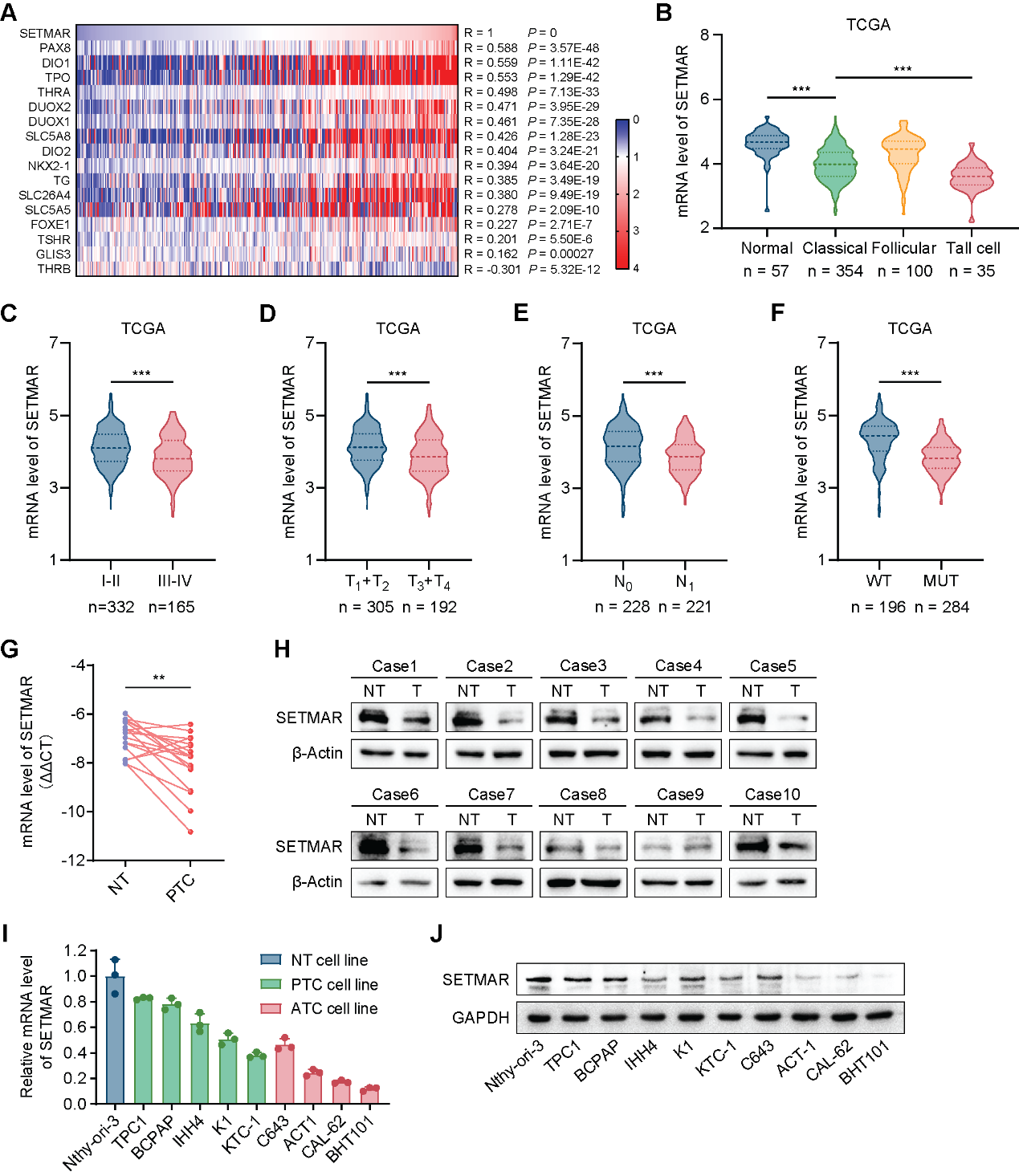
 **Figure S1**

(A) The correlation between SETMAR expression and TDS-related genes expression in the TCGA database was illustrated by heat map.

(B) The expression analysis of SETMAR in different subtypes of thyroid cancer and normal thyroid tissue in the TCGA database.

(C, D, E, F) The correlation analysis of SETMAR expression with the patient's clinical stage (C), tumor size (D), lymph node metastasis (E), and BRAF mutation (F) in patients with papillary thyroid carcinoma in the TCGA database.

(G) The relative mRNA expression levels of SETMAR were assessed by RT-qPCR in 16 paired normal thyroid tissues and PTC tissues, relative SETMAR expression data was normalized with β-actin and represented on a log2 scale. The difference was analyzed using paired *t* tests (***P* < 0.01).

(H) The relative protein expression levels of SETMAR were assessed by western blotting in 10 paired normal thyroid tissues and PTC tissues.

(I, J) The mRNA (I) and protein (J) expression levels of SETMAR were assessed by RT-qPCR and Western blotting respectively in a cohort of thyroid cancer cell lines and a normal thyroid cell line.

Data are shown as the median with range in (B, C, D, E, and F) and the mean ± SD of three replicates in (I). *P* values were determined using two-tailed unpaired Student’s *t*-test (****P* < 0.001).


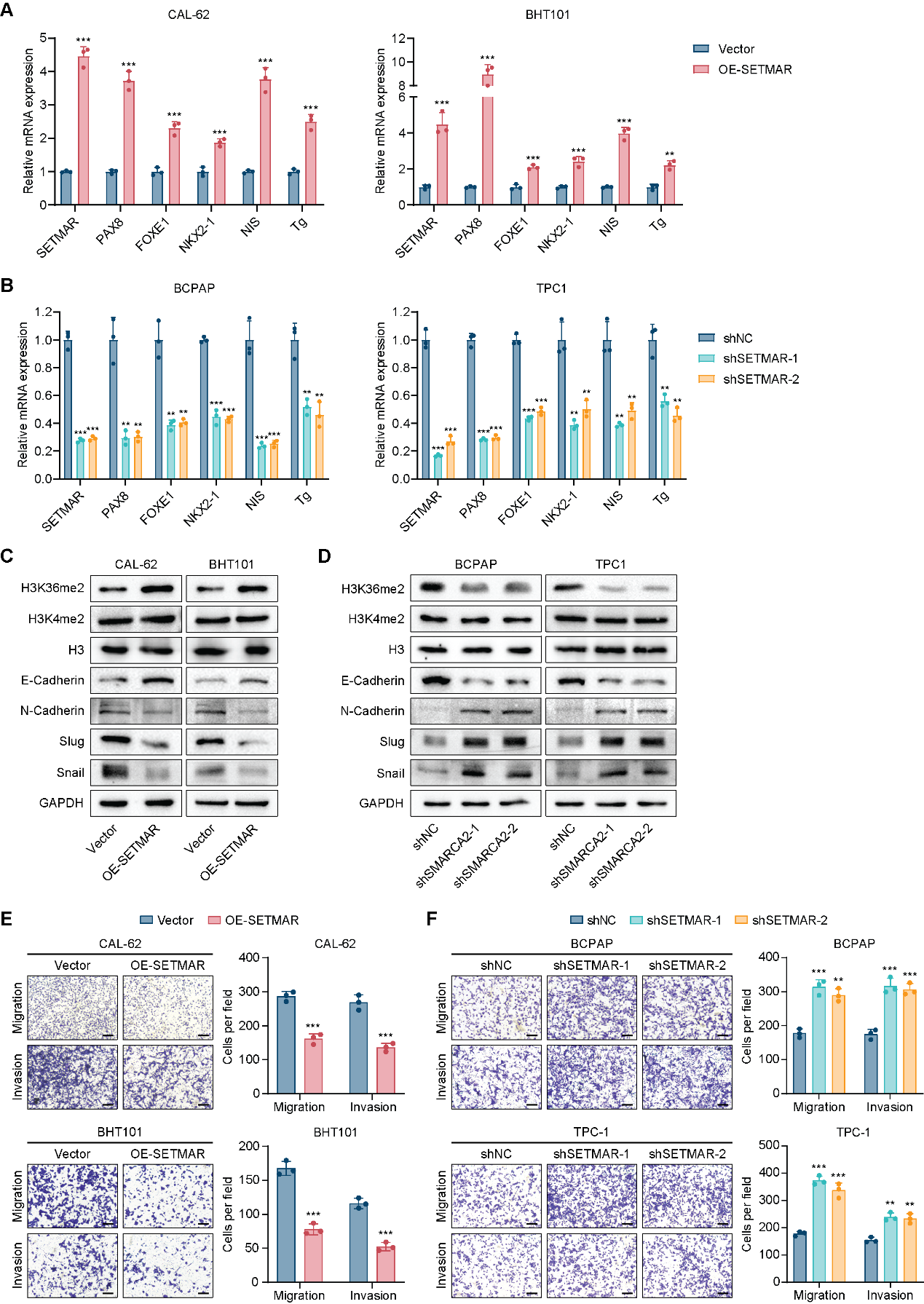


**Figure S2**

(A, B) RT-qPCR was performed to measure the mRNA expression of SETMAR and thyroid differentiation markers in ATC cells with or without SETMAR overexpression (A) and PTC cells with or without SETMAR knockdown (B).

(C, D) Western blot analysis of total H3K4me2, total H3K36me2 levels and EMT-related markers, including E-cadherin, N-cadherin, Slug, and Snail, in ATC cells after SETMAR overexpression (C) and PTC cells after SETMAR knockdown (D).

(E, F) The cell migration and invasion abilities of ATC cells following SETMAR overexpression (E) and PTC cells following SETMAR knockdown (F) were determined by Transwell assay. The scale bar is 200 μm.

Data are shown as the mean ± SD of three replicates in (A, B, E, and F). *P* values were determined using two-tailed unpaired Student’s *t*-test (***P* < 0.01, ****P* < 0.001).


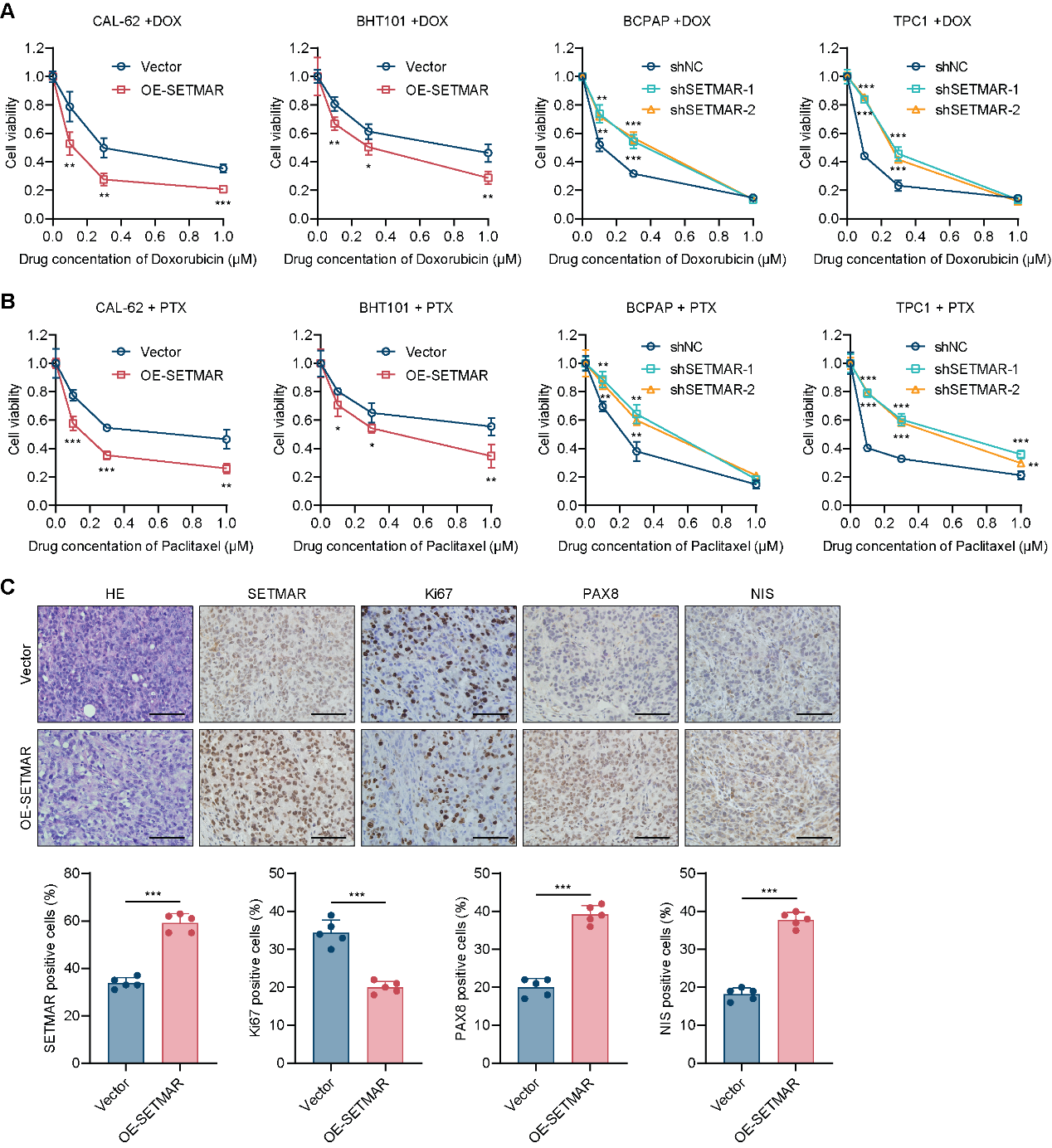


**Figure S3**

(A, B) CCK-8 assay was conducted to measure the cell viability of SETMAR overexpressed ATC cells and SETMAR-silencing PTC cells following treatment with doxorubicin (A) or paclitaxel (B) in different concentration for 48 hours.

(C) Expression levels of SETMAR, Ki67, PAX8, and NIS in xenografts generated from CAL-62 cells with or without SETMAR overexpression were assessed by immunohistochemical staining. An unpaired two-tailed Student's t-test was used to analyze the difference in the percentage of positively stained cells between the two groups. Scale bar is 50 μm.

Data are shown as the mean ± SD of three replicates in (A, B) and five replicates in (C). *P* values were determined using two-tailed unpaired Student’s *t*-test (**P* < 0.05, ***P* < 0.01, ****P* < 0.001).


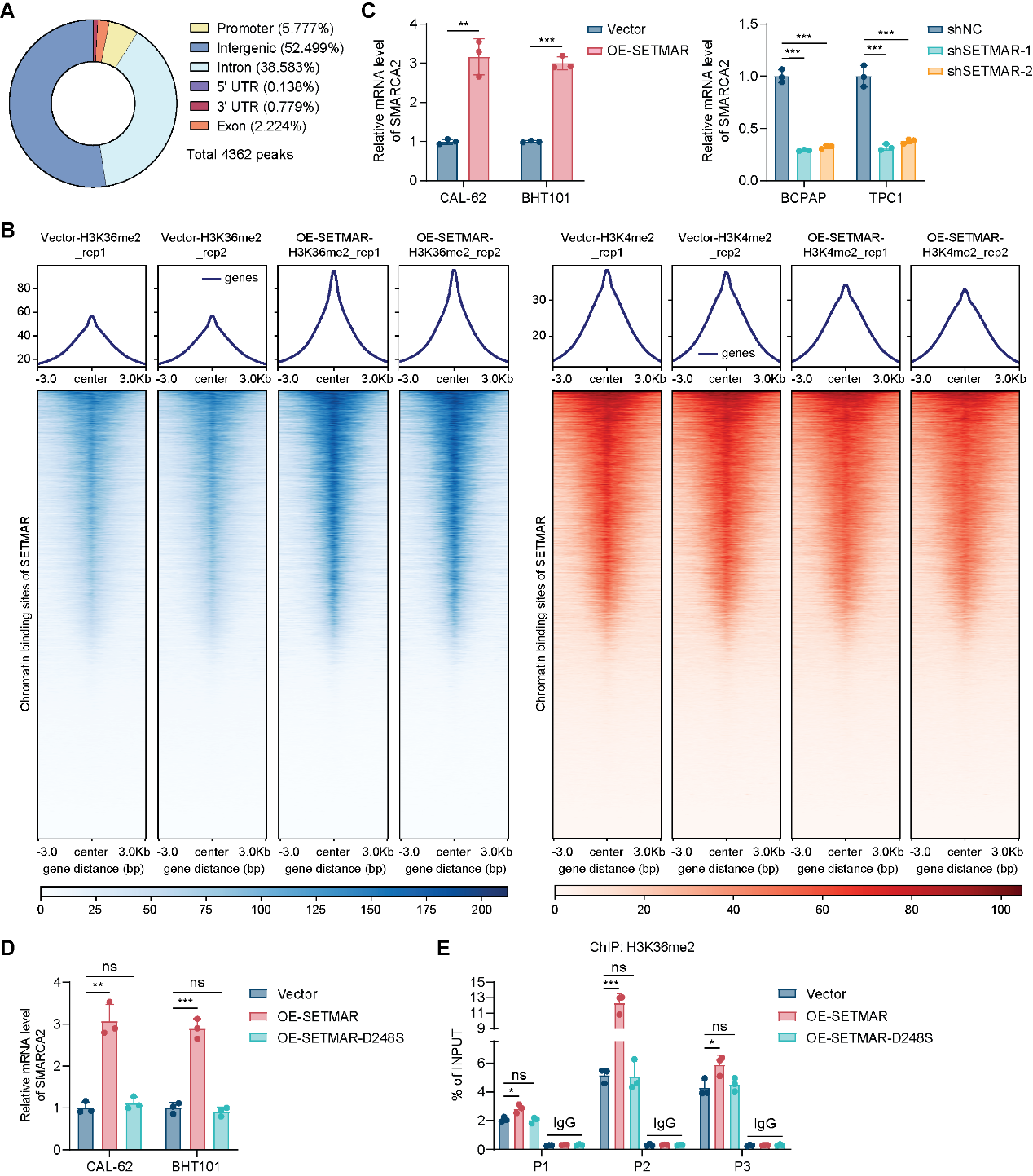


**Figure S4**

(A) Distribution of 4362 SETMAR binding sites in the genome based on SETMAR-3×Flag CUT&TAG peak maps.

(B) Heatmap of the H3K36me2 and H3K4me2 CUT&TAG signal within SETMAR-3×Flag peaks in CAL-62 cells with or without SETMAR overexpression. The peaks were sorted according to the SETMAR-3×Flag signal in SETMAR-3×Flag overexpression cells.

(C) RT-qPCR was performed to assess the alterations in SETMAR expression following SETMAR overexpression or knockdown.

(D) RT-qPCR was utilized to measure the mRNA expression of SMARCA2 in SETMAR-overexpressing, SETMAR-D248S-overexpressing, and control CAL-62 cells.

(E) The enrichment levels of H3K36me2 in different regions (chr9:2015649–2013770, P1; chr9:2014750–2014823, P2; chr9:2015618–2015688, P3) of the SMARCA2 gene promoter were determined by ChIP‒qPCR in SETMAR-overexpressing, SETMAR-D248S-overexpressing, and control CAL-62 cells.

Data are shown as the mean ± SD of three replicates in (C, D, and E). *P* values were determined using two-tailed unpaired Student’s *t*-test (**P* < 0.05, ***P* < 0.01, ****P* < 0.001. ns, no significance).


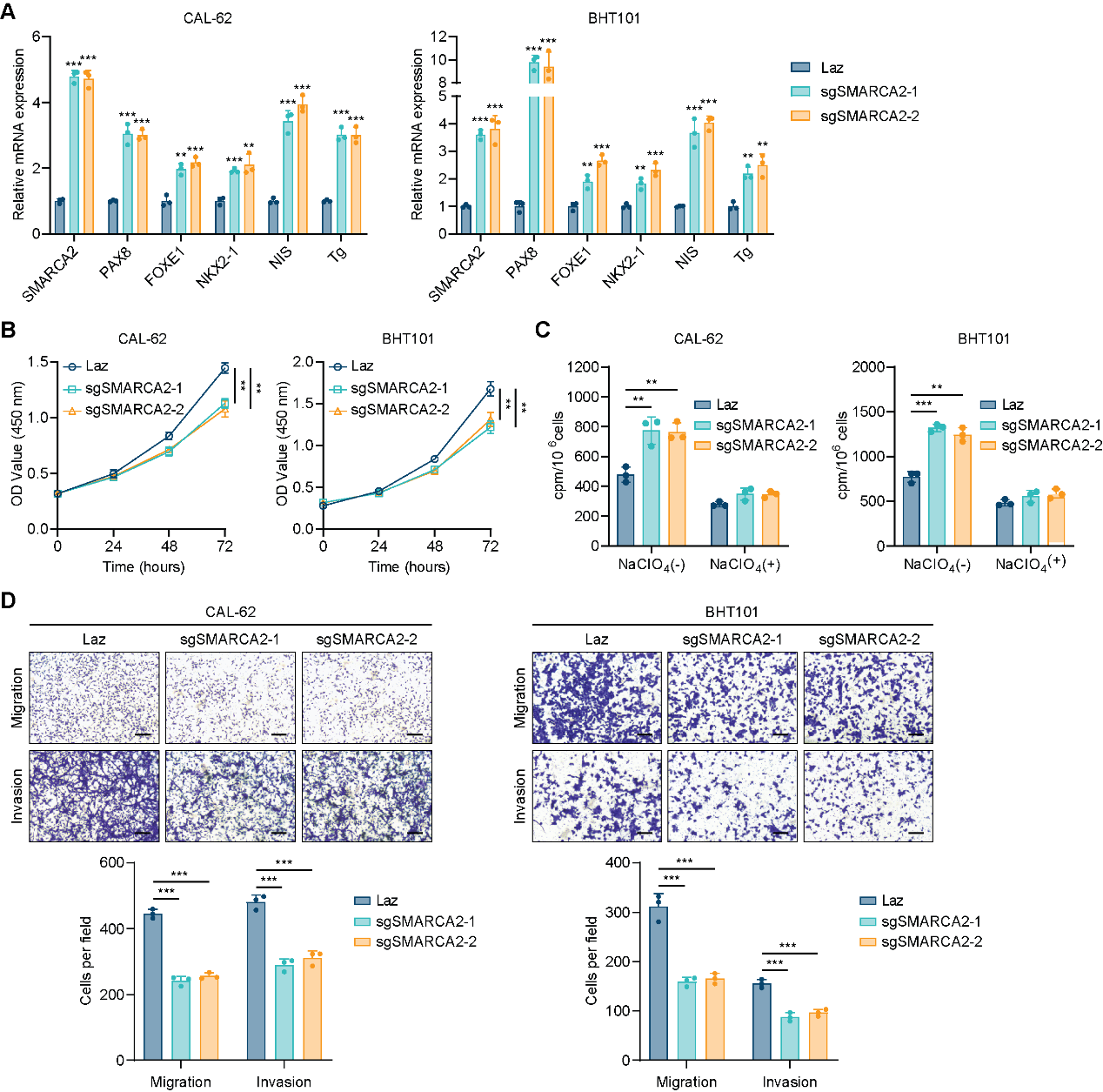


**Figure S5**

(A) RT-qPCR was performed to quantify the mRNA expression of SMARCA2 and thyroid differentiation markers in ATC cells with or without SETMAR overexpression.

(B) CCK-8 assay was performed to measure the cell proliferation of ATC cells with or without SMARCA2 overexpression.

(C) Radioactive iodine uptake assay was utilized to determine the influence of SMARCA2 overexpression on the ability to uptake radioactive iodine of ATC cells.

(D) Transwell assay determined the cell migration and invasion abilities of ATC cells following SMARCA2 overexpression. The scale bar is 200 μm.

Data are shown as the mean ± SD of three replicates in (A, B, C, and D). *P* values were determined using two-tailed unpaired Student’s *t*-test (**P* < 0.05, ***P* < 0.01, ****P* < 0.001).


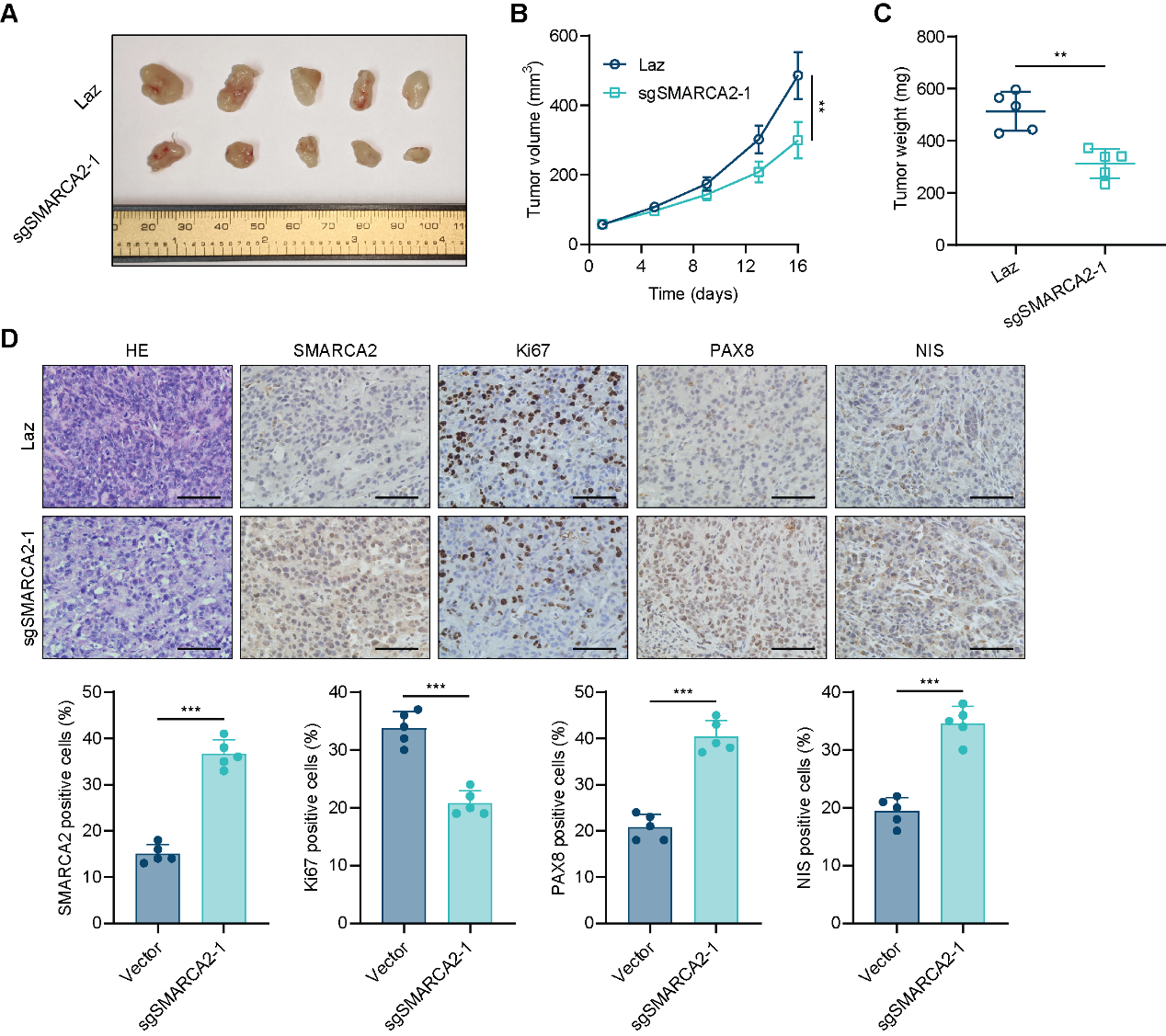


**Figure S6**

(A) Representative images of subcutaneous xenografts in nude mice derived from CAL-62 cells with or without SMARCA2 overexpression.

(B) Growth curves of the subcutaneous xenografts in SMARCA2-overexpressed group and control group.

(C) Analysis of the tumor weight of the xenografts in SMARCA2-overexpressed group and control group.

(D) Expression levels of SMARCA2, Ki67, PAX8, and NIS in xenografts generated from CAL-62 cells with or without SMARCA2 overexpression were assessed by immunohistochemical staining. An unpaired two-tailed Student's t-test was used to analyze the difference in the percentage of positively stained cells between the two groups. Scale bar is 50 μm.

Data are shown as the mean ± SD of five replicates in (B, C, and D). *P* values were determined using two-tailed unpaired Student’s *t*-test (***P* < 0.01, ****P* < 0.001).


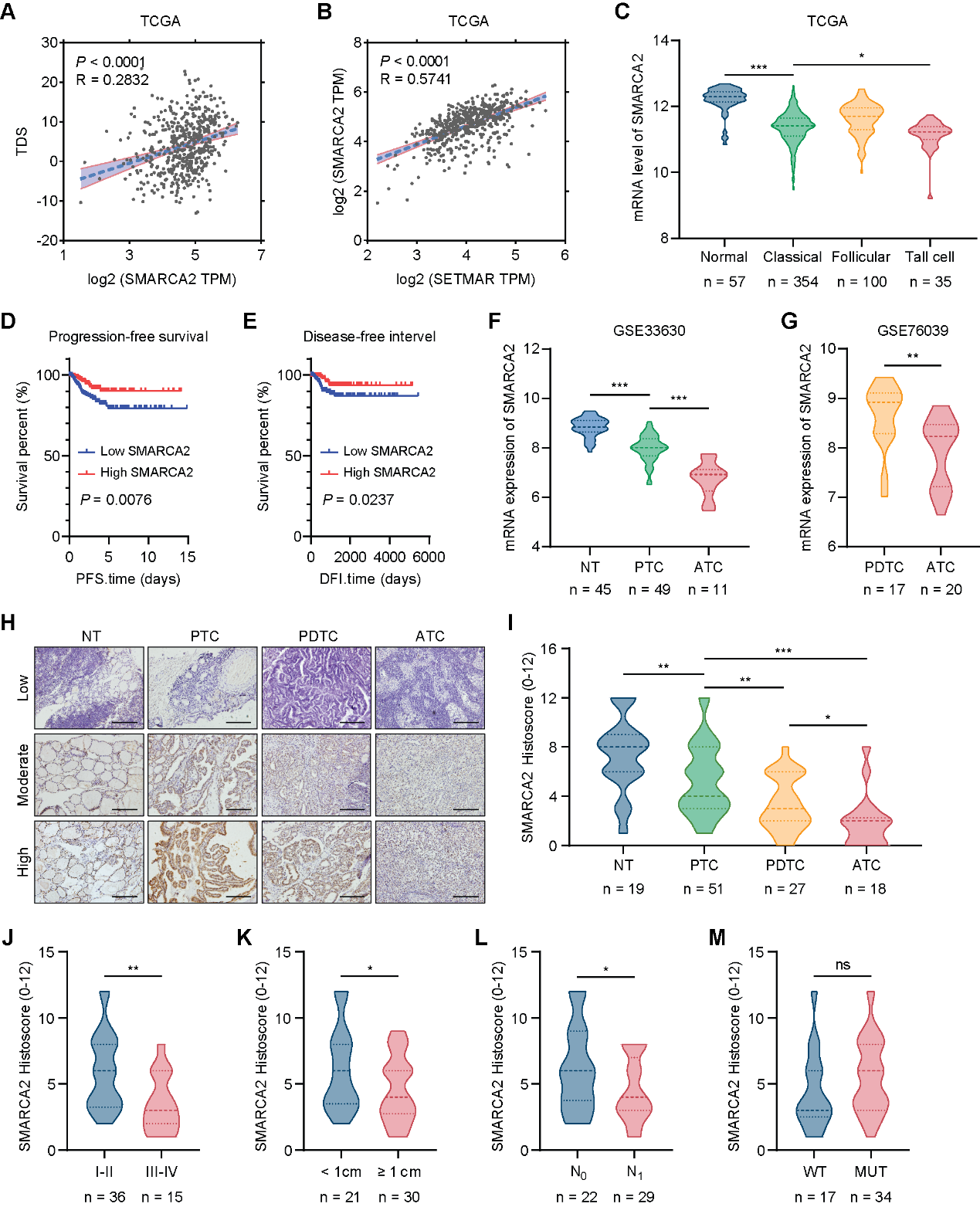


**Figure S7**

(A) Pearson’s correlation analysis of SMARCA2 expression and TDS levels in the TCGA database.

(B) Pearson’s correlation analysis of SMARCA2 expression and SETMAR expression in the TCGA database.

(C) Expression analysis of SMARCA2 in different subtypes of thyroid cancer and normal thyroid tissue in the TCGA database.

(D, E) Kaplan–Meier survival analysis showed that low SMARCA2 expression was significantly associated with poor patient disease-free survival (D) and poor progression-free survival (E) in the TCGA database (*P* values were determined by log-rank test).

(F, G) Expression analysis of SMARCA2 in different types of thyroid cancer in GSE33630 (F) and GSE70639 (G).

(H, I) Immunohistochemical staining of SMARCA2 was performed in normal thyroid (NT) tissue, papillary thyroid carcinoma (PTC) tissue, poorly differentiated thyroid carcinoma (PDTC) tissue, and anaplastic thyroid carcinoma (ATC) tissue. The differences in expression across cancer subtypes were analyzed.

(J, K, L, M) The correlation analysis of immunohistochemical staining score of SMARCA2 with the patient's clinical stage (J), tumor size (K), lymph node metastasis (L), and BRAF mutation (M) in patients with papillary thyroid carcinoma.

Data are shown as the median with range in (C, F, G, I, J, K, L, and M). *P* values were determined using two-tailed unpaired Student’s *t*-test (**P* < 0.05, ***P* < 0.01, ****P* < 0.001. ns, no significance).


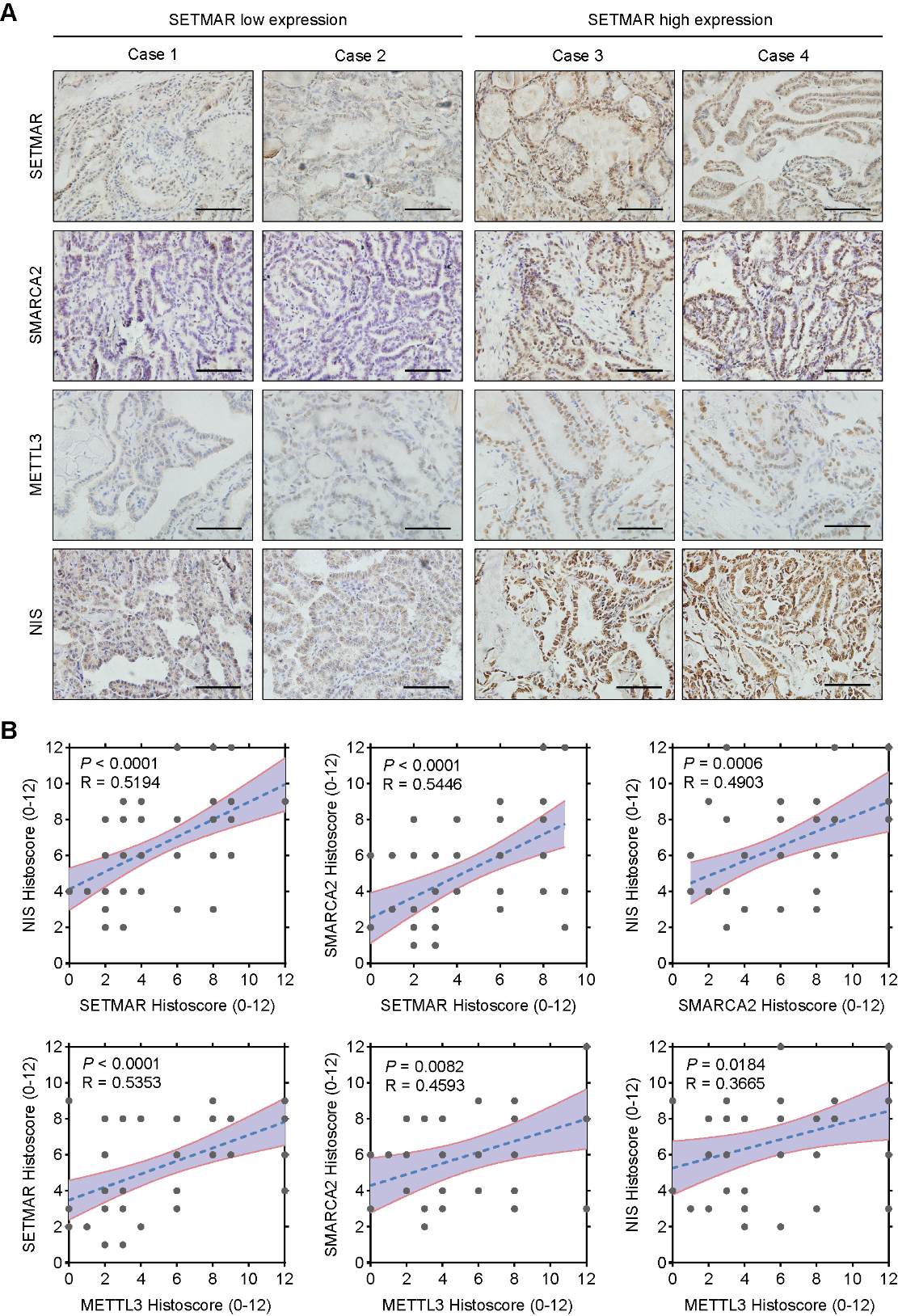


**Figure S8**

(A) Representative immunohistochemical images of SETMAR, SMARCA2, METTL3 and NIS staining in PTC tissues. Scale bar is 50 μm.

(B) Analysis of the Pearson’s correlation in histoscore of immunohistochemical staining among SETMAR, SMARCA2, METTL3 and NIS.


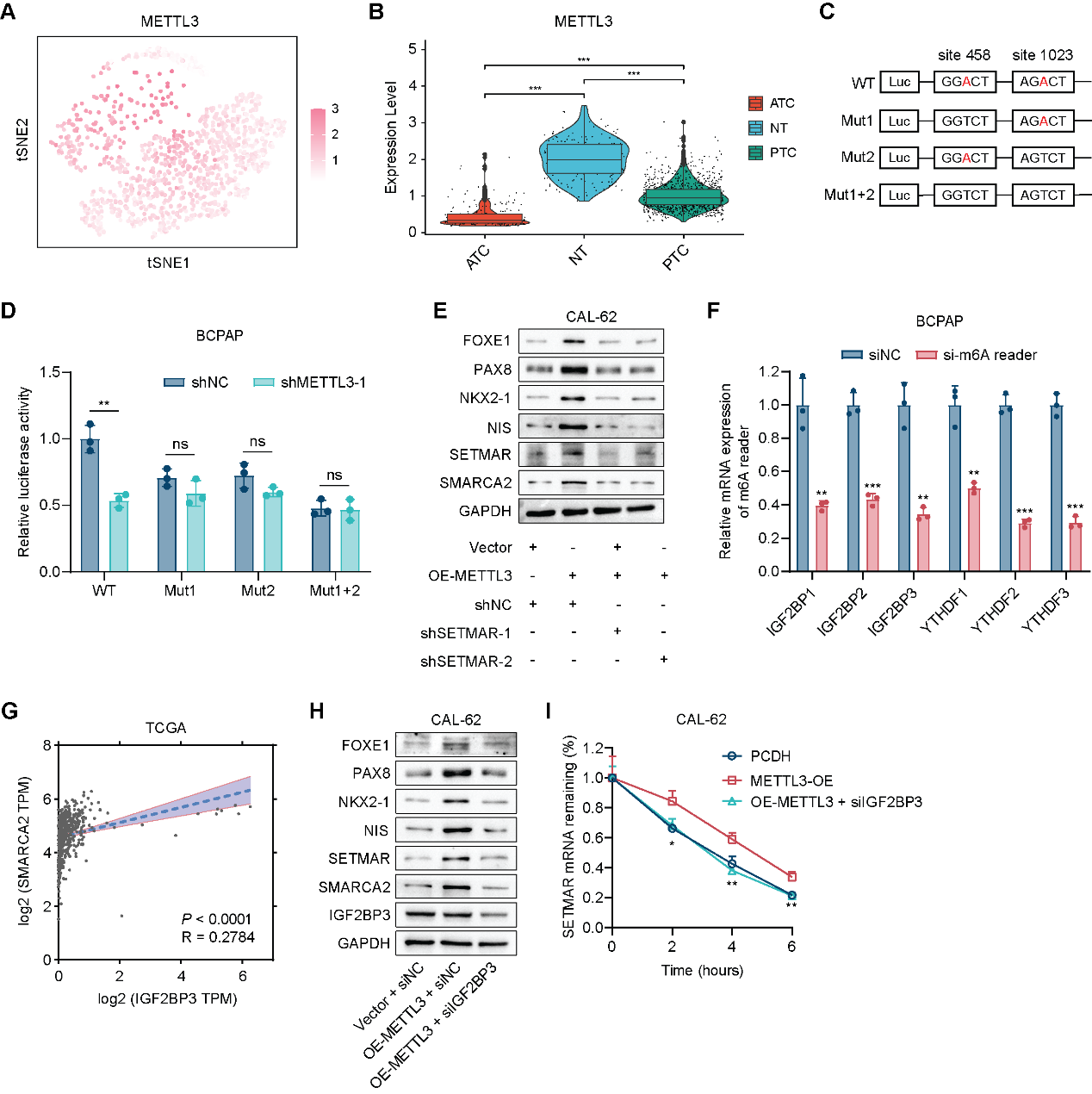


**Figure S9**

(A, B) Analysis of METTL3 expression in thyroid follicular cells, PTC, and ATC cells in the integrated single cell sequencing dataset.

(C) Wild-type or m6A consensus sequence mutant SETMAR cDNA was fused with firely luciferse reporter.

(D) The METTL3 silenced BCPAP cells and control BCPAP cells were transfected with WT and mutant SETMAR (Mut1, Mut2, and Mut1+2), and the Dual-luciferase reporter assay was employed to assess the luciferase activity across all experimental groups.

(E) The expression of SMARCA2 and thyroid differentiation markers was measured in METTL3-overexpressing CAL-62 cells before and after SETMAR knockdown using western blotting.

(F) The effect of siRNA on the silencing of m6A “readers” was validated by RT-qPCR.

(G) Pearson’s correlation analysis of IGF2BP3 expression and SMARCA2 expression in the TCGA database.

(H) Western blotting was used to detect the protein expression of SETMAR, SMARCA2 and thyroid differentiation markers in METTL3 overexpressing ATC cells following IGF2BP3 silencing.

(I) RT-qPCR was utilized to measure the mRNA stability of SETMAR in METTL3 overexpressing ATC cells following IGF2BP3 silencing.

Data are shown as the median with range in (B) and the mean ± SD of three replicates in (D, F, and I). *P* values were determined using two-tailed unpaired Student’s *t*-test (**P* < 0.05, ***P* < 0.01, ****P* < 0.001. ns, no significance).


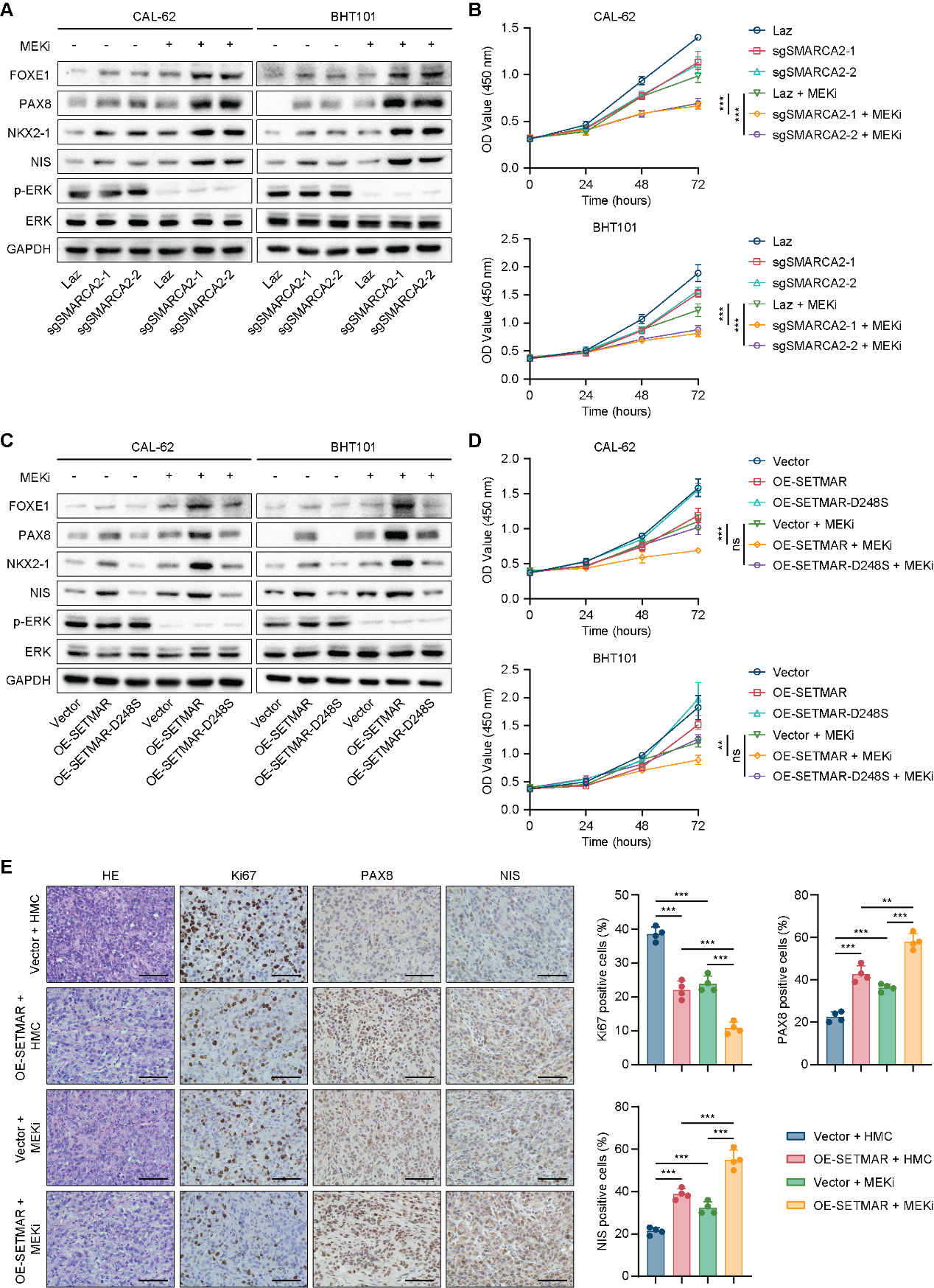


**Figure S10**

(A) Western blotting was utilized to detect the expression of thyroid differentiation markers in ATC cells with SMARCA2 overexpression before and after Selumetinib treatment (1 μM) for 24 hours.

(B) CCK-8 assay was performed to determine the proliferation of SMARCA2 overexpressing ATC cells with Selumetinib treatment (1 μM) or not.

(C) Western blotting was used to detect the expression of thyroid differentiation markers in wild-type SETMAR or SETMAR-D248S overexpressing ATC cells before and after treatment with selumetinib (1 μM) for 24 hours.

(D) CCK-8 assay was used to detect the proliferation of wild-type SETMAR-overexpressed and SETMAR-D248S-overexpressed ATC cells with Selumetinib treatment (1 μM) or not.

(E) Expression levels of SMARCA2, Ki67, PAX8, and NIS in xenografts generated from CAL-62 cells with or without SETMAR overexpression following Selumetinib treatment were assessed by immunohistochemical staining. An unpaired two-tailed Student's t-test was used to analyze the difference in the percentage of positively stained cells between the two groups. Scale bar is 50 μm.

Data are shown as the mean ± SD of three replicates in (B, D) and four replicates in (E). *P* values were determined using two-tailed unpaired Student’s *t*-test (***P* < 0.01, ****P* < 0.001. ns, no significance).


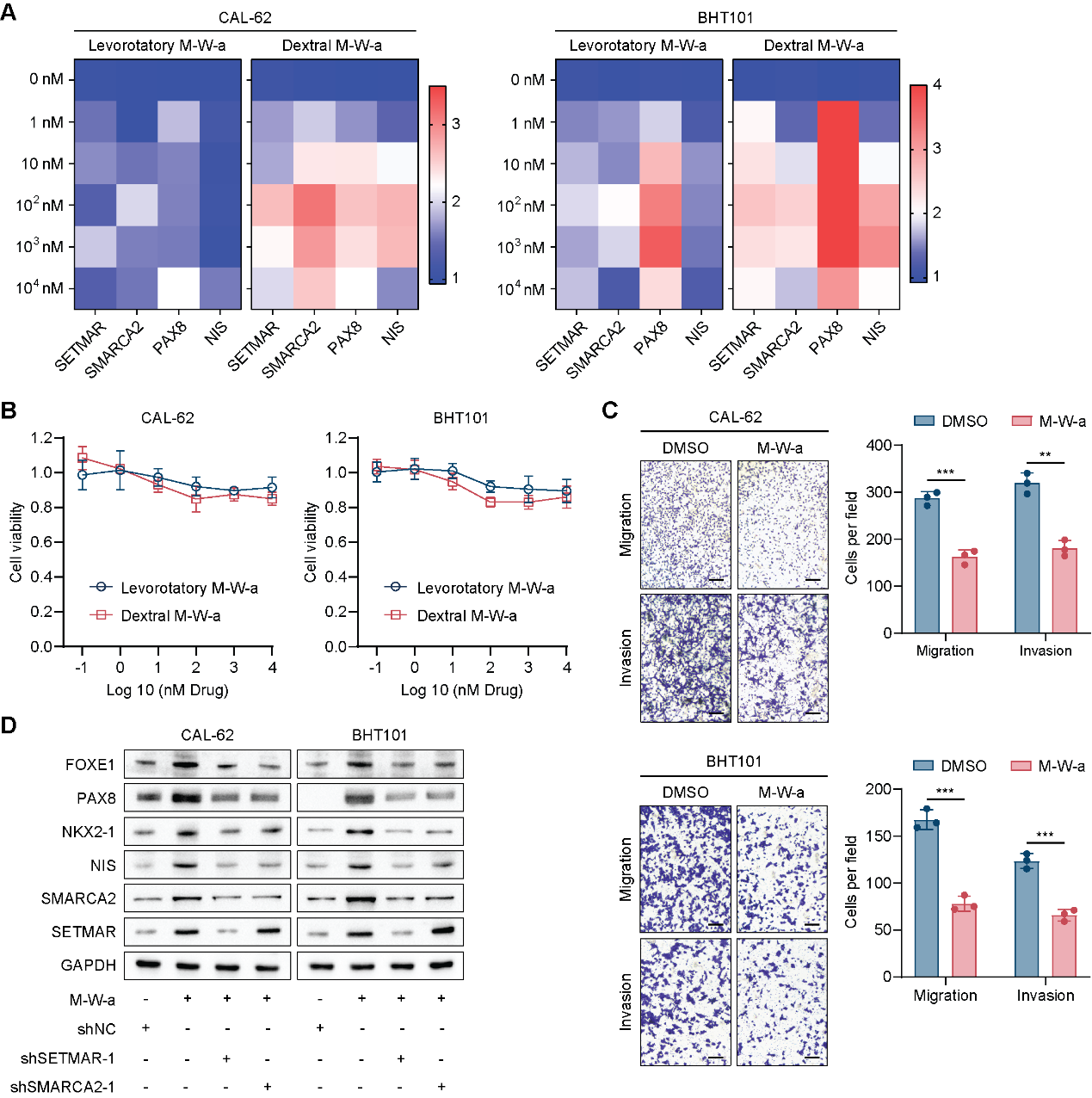


**Figure S11**

(A) RT-qPCR was performed to measure the mRNA expression of SETMAR, SMARCA2, PAX8 and NIS in ATC cells following the treatment of levorotatory or dextral METTL3-14-WTAP activator in divergent concentrations for 24 hours.

(B) CCK8-assay was used to determine the cell viability of ATC cells after the treatment of levorotatory or dextral METTL3-14-WTAP activator in divergent concentrations for 48 hours.

(C) Transwell assay determined the cell migration and invasion abilities of ATC cells following the treatment of dextral METTL3-14-WTAP activator in 100nM for 24 hours. The scale bar is 200 μm.

(D) Western blotting was conducted to confirm the on-target effects of METTL3-14-WTAP activator by detect the expression of thyroid differentiation markers in METTL3-14-WTAP activator-treated ATC cells before and after SETMAR or SMARCA2 knockdown.

Data are shown as the mean ± SD of three replicates in (B, C). *P* values were determined using two-tailed unpaired Student’s *t*-test (***P* < 0.01, ****P* < 0.001).


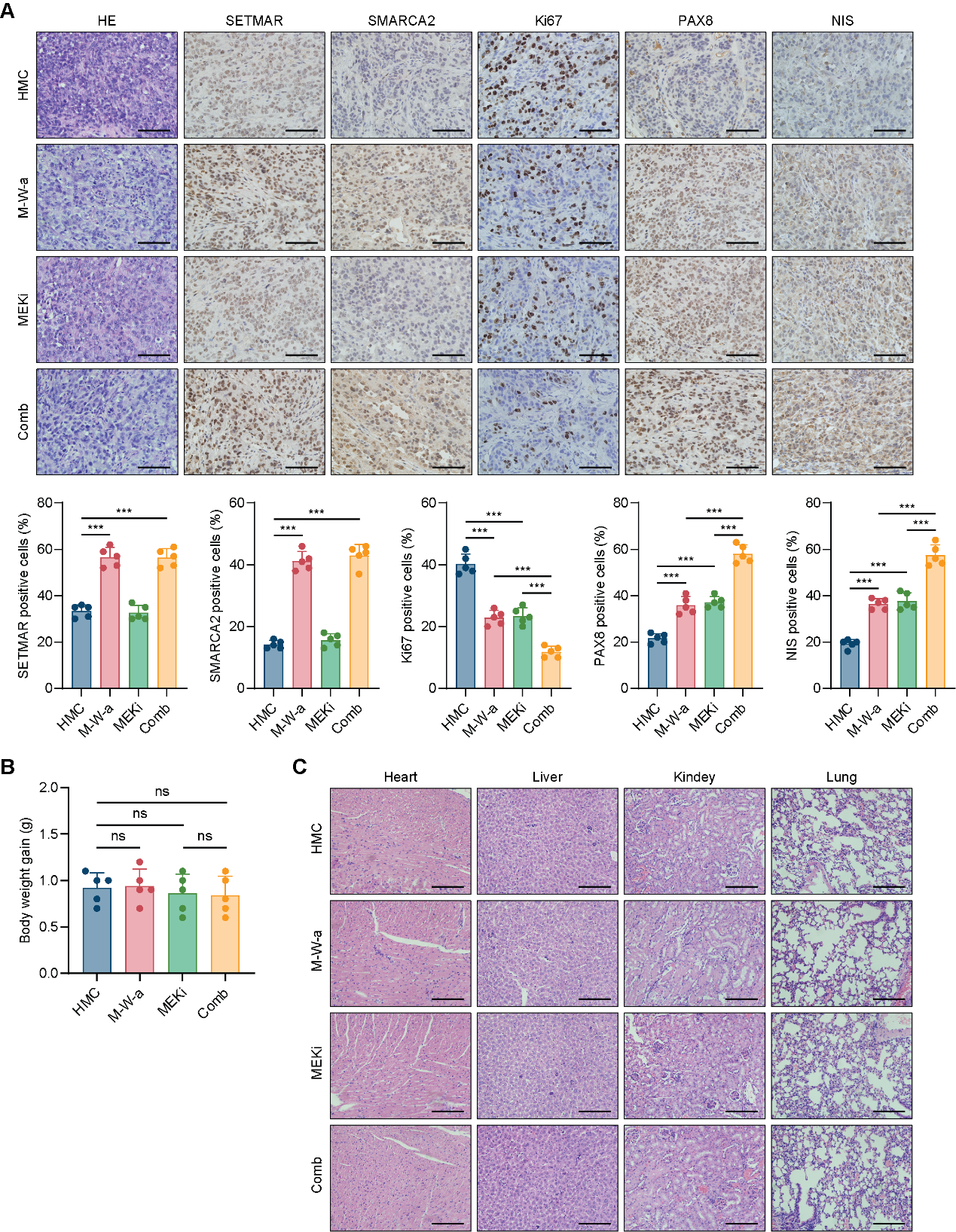


**Figure S12**

(A) The expression levels of SETMAR, SMARCA2, PAX8, NIS and Ki67 in xenografts of each group were assessed by immunohistochemical staining. An unpaired two-tailed Student's *t*-test was used to analyze the difference in the percentage of positively stained cells between the two groups. Scale bar is 50 μm.

(B) The alteration in body weight in each group of mice after treatment.

(C) H&E staining of lung, liver, heart, and kidney tissue of nude mice in each group.

Data are shown as the mean ± SD of five replicates in (B, C). *P* values were determined using two-tailed unpaired Student’s *t*-test (****P* < 0.001. ns, no significance).

Additional File 1

1.Primers for RT-qPCR

| Name | Forward Primer (5'-3') | Reverse Primer (5'-3') |
| --- | --- | --- |
| β-actin | GATCATTGCTCCTCCTGAGC | ACTCCTGCTTGCTGATCCAC |
| SETMAR | GAAAGGGCCCAATTCTTCTC | AGCATTTTCTGCATCCTGCT |
| PAX8 | AAGGTGGTGGAGAAGATTGG | AGGGAGGTTGAATGGTTGC |
| NKX2-1 | GTACCAGGACACCATGAGGAAC | CCATGTTCTTGCTCACGTC |
| FOXE1 | AACCCCAAAAAGTGGCAGAA | GCGGGATCTTGAGGAAGCA |
| Tg | CTGGCTGAGACAGGTTTGGA | GACTGATTGAACTGCGAGGAA |
| NIS | GTTCTACACTGACTGCGACCCTC | GCAGCCGAGGTTTGATGAG |
| SMARCA2 | GCAGCCGAGGTTTGATGAG | TGCCTGAAGATTTAAGCCCAG |
| METTL3 | CTTCAGCAGTTCCTGAATTAGC | ATGTTAAGGCCAGATCAGAGAG |
| IGF2BP1 | AGTGGAATTGCATGGGAAAATCA | CAACGGCGGTTTCTGTGTC |
| IGF2BP2 | AGTGGAATTGCATGGGAAAATCA | CAACGGCGGTTTCTGTGTC |
| IGF2BP3 | GCACTTCCCTTTGTTGTAGTC | AGCACTTCCCTTAGGTTACTC |
| YTHDF1 | ACCTGTCCAGCTATTACCCG | TGGTGAGGTATGGAATCGGAG |
| YTHDF2 | GTTGGTAGCGGGTCCATTACT | GGTCTTCAGTTTAGGTTGCTGT |
| YTHDF3 | GGTGTATTTAGTCAACCTGGGG | AAGAGAACTAGGTGGATAGCCAT |

2. Sequences of shRNA, siRNA and sgRNA

| Name | Sequence (5'-3') |
| --- | --- |
| shSETMAR-1 | CGCAACATCAACAATGCATTT |
| shSETMAR-2 | CAAGTGTTCAAGACGCATAAA |
| shSMARCA2-1 | CCAAACCTGTAGTGAGCGATT |
| shSMARCA2-2 | GCTGAGAAACTGTCACCAAAT |
| shMETTL3-1 | GGAACATTATGATCCAGAAAC |
| shMETTL3-2 | GGAACTGGATCCTACCTATGT |
| siNC | UUCUCCGAACGUGUCACGUTT |
| siIGF2BP1 | AAGCUGAAUGGCCACCAGUUG |
| siIGF2BP2 | GCGAAAGGAUGGUCAUCAU |
| siIGF2BP3 | CCUUGAAAGUAGCCUAUAUTT |
| siYTHDF1 | CCUGCUCUUCAGCGUCAAUTT |
| siYTHDF2 | GGUGAAGCUGCUUGGUCUA |
| siYTHDF3 | TAAGTCAAAGAAGACGTATTA |
| Laz | CCCGAATCTCTATCGTGCGG |
| sgSMARCA2-1 | CGGAGCCCGAGTTTAGGAAG |
| sgSMARCA2-2 | GCTGGGCGGAGCCCGAGTTT |

1. Primers for ChIP-qPCR

| Name | Forward Primer (5'-3') | Reverse Primer (5'-3') |
| --- | --- | --- |
| SMARCA2-promoter P1 | TCACCAACACTCCCAGCCATC | AGAACGCCAAATACTGCCATGTAG |
| SMARCA2-promoter P2 | CCCGTCCCCTCCCTCTCTC | TGGAGAAGGTGCTGGAATTTAACTG |
| SMARCA2-promoter P3 | CAGGTGGAATGCTCTAACGACAG | GCAAGGTTCGGTTGAAACTTTCTC |
| PAX8-enhancer | CCAGGGTCTCCTTCCAGAAGCC | CCAGTTGGTTTGGGTAGGGATGTG |
| FOXE1-enhancer | GCGGAGGAAGAACAAGAGACAAC | ACTGTGTGGCTGGTGAGATAGAC |

1. Primers for Chromosome Conformation Capture (3C) assay

| Name | Forward Primer (5'-3') | Reverse Primer (5'-3') |
| --- | --- | --- |
| PAX8 | AAGAAAGGAGAGGGTCCCAGGA | CAACACCCAGTGAAGCCAGG |
| FOXE1 | GCCCCTCTCCCTTCGTTCG | TGAAATCACTGCAGCTTCCTGT |

1. Primers for MeRIP-qPCR

| Name | Forward Primer (5'-3') | Reverse Primer (5'-3') |
| --- | --- | --- |
| Primer 458 | GGTCCAGAAAGGTCTACAGTTCCA | ACCTCACCAGCATATTCACAGACA |
| Primer 1023 | CCCCTTCTGTGTTCCCCTCCTG | GCGAGTTGTTTCTGCTGCTTTACG |

1. Antibodies and drugs used in this study

| Antibodies name | Application | Supplier | Cat # |
| --- | --- | --- | --- |
| Anti-human SETMAR antibody | IHC | ABclonal Technology | A6738 |
| Anti-human SETMAR antibody | WB | Santa Cruz Biotechnology | sc-515243 |
| Anti-human PAX8 antibody | WB, IHC | BOSTER Biological Technology | BM4807 |
| Anti-human NKX2-1 antibody | WB | Bimake | A5549 |
| Anti-human FOXE1 antibody | WB | Santa Cruz Biotechnology | sc-518211 |
| Anti-human NIS antibody | WB, IHC | Proteintech | 24324-1-AP |
| Anti-human H3K36me2 antibody | WB, ChIP, CUT&Tag | Abcam | Ab9049 |
| Anti-human H3K4me2 antibody | WB, ChIP, CUT&Tag | Cell Signaling Technology | 9725 |
| Anti-human METTL3 antibody | WB | Abcam | ab195352 |
| Anti-human SMARCA2 antibody | WB, ChIP, IHC | Cell Signaling Technology | 11966 |
| Anti-human E-Cadherin antibody | WB | Cell Signaling Technology | 3195 |
| Anti-human E-Cadherin antibody | WB | Cell Signaling Technology | 13116 |
| Anti-human Snail antibody | WB | Cell Signaling Technology | 3879 |
| Anti-human Slug antibody | WB | Cell Signaling Technology | 9585 |
| Anti-human β-actin antibody | WB | ABclonal Technology | AC026 |
| Anti-human H3 antibody | WB | Cell Signaling Technology | 4499 |
| Anti-human Ki67 antibody | IHC | Cell Signaling Technology | 9449 |
| Anti-human IGF2BP3 antibody | WB, RIP | Medical & Biological Laboratories | RN009P |
| Anti-m6A antibody | MeRIP | Synaptic Systems | 202 003 |
| Anti-human H3K27ac antibody | ChIP | Abcam | Ab4729 |
| Anti FLAG antibody | WB, ChIP, CUT&Tag | Cell Signaling Technology | 14793 |
| Anti-human GAPDH antibody | WB | Santa Cruz Biotechnology | sc-47724 |
| Goat Anti-Rabbit IgG (HRP) | WB | ABclonal Technology | AS014 |
| Normal Rabbit IgG | ChIP, CUT&Tag | Cell Signaling Technology | 2729 |
| Actinomycin D | / | Selleck | S8964 |
| Puromycin 2HCl | / | Selleck | S7417 |
| Blasticidin S | / | Solarbio | B9300 |
| Hygromycin B | / | Solarbio | H8080 |
| Doxorubicin | / | Selleck | S1208 |
| Paclitaxel | / | Selleck | S1150 |
| Selumetinib | / | Selleck | S1008 |

7. The METTL3-14-WTAP activator was synthesized by School of Pharmacy, Tianjin Medical University. The NMR spectrum result of this compound is as follows:


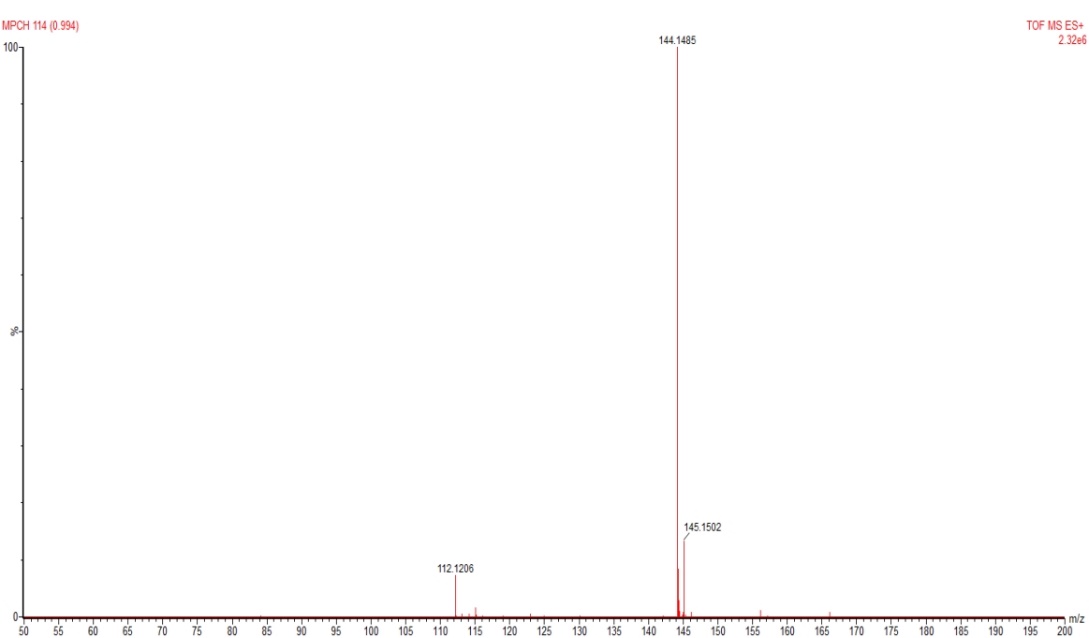


**Additional file 2**

*Thyroid Differentiation Score (TDS) caculation*

TDS classifies samples based on the mRNA expression levels of 16 genes selected for thyroid function, including TG, TSHR, TPO, PAX8, FOXE1, SLC26A4, DIO1, DUOX2, NKX2-1, DIO2, GLIS3, SLC5A5 (NIS), THRA, THRB, DUOX1 and SLC5A8. Normalized RSEM log2 values ​​were first clustered around the sample median to obtain log2 (fold change) and then summed over the 16 genes in each sample: TDS = mean of the 16 log2 genes (fold change).

*Analysis of single-cell datasets*

Single cell data from a public database was used in this study. ​The ATC data was obtained from GSE148673, the PTC data from GSE184362 and the normal cells from GSE134355. The Seurat algorithm was applied to filter out cells with more than 2500 or fewer than 200 genes. In addition, cells with more than 20% mitochondria and more than 5% HB genes were excluded, as were cells with total RNA counts below 100000. This resulted in a final dataset of 66,061 cells and 42,097 genes. The Harmony algorithm is then used to integrate out the batch effects. Normalization was performed using the 'LogNormalize' method with a 10, 000-fold scaling. Subsequently, 3,000 hypervariable genes were selected for principal component analysis (PCA) to reduce dimensionality. The optimal number of principal components was determined using an Elbowplot. The SNN algorithm is used for clustering and the TSNE algorithm is used for visualization. The SingleR algorithm was employed in this study for cell annotation and extraction of thyroid epithelial cells. To validate the classification, the AddModuleScore algorithm is used to visualize cell classification based on TDS scores.

*Immunohistochemistry (IHC)*

​Tumor tissue sections were stained with the indicated antibodies using a standard immunohistochemistry protocol. The signal was visualized with the DAB Substrate Kit (ZLI-9017, Zsbio, China). The histoscore were calculated by two experienced pathologists as follows: histoscore = staining intensity × percentage of positive tumor cells. The staining intensity was regarded as 0 (no staining); 1 (weak, light yellow); 2 (moderate, light brown); or 3 (intense, brown). The percentage of positive cells was regarded as 0 (<5%), 1 (5–25%), 2 (25–50%), 3 (51–75%), or 4 (>75%).

*Cell viability assay*

​A total of 1000 to 2000 cells are seeded in each well of a 96-well plate. Cell viability was measured using Cell Counting Kit-8 (CCK-8) (C0038, Beyotime, China) following the provided instructions.

*Transwell migration and invasion assay*

Cells were harvested and seeded on the upper layer of transwell chamber (Corning) at a density of 1.5 × 10^4^ cells per chamber for migration assay. The culture medium without fetal bovine serum was added for cell culture, while normal cell culture medium was added in the lower layer. After 24 hours of continued culture, the cells were fixed with paraformaldehyde and stained with 0.1% crystal violet. Three randomly selected fields of view were examined under a microscope and the stained cells were counted. For the invasion assay, cells were seeded on the upper layer of a Matrigel-coated transwell chamber and cultured for 48 hours.

*Luciferase reporter assay of SETMAR m6A sites*

Wild-type and m6A consensus sequence mutant SETMAR cDNA were synthesized by (Hanbio, China) and fused with firely luciferse reporter. The adenosine (A) in the m6A motif was replaced by thymine (T). ATC cells were seeded into 24-well plate followed by co-transfection of 0.5 μg of wild-type or mutated SETMAR reporter plasmids and 20 ng renilla luciferase reporter vector. After 24–36 h, cells were harvested to access the luciferase activity using Dual-Glo Luciferase system (Promega) with the normalization to pRL-TK. Each group was conducted in triplicate.

*RIP-qPCR*

The cells were cross-linked with 1% formaldehyde and treated with a 150 mM NaCl RIPA buffer containing RNase and protease inhibitors. Subsequently, the cells were lysed in a solution consisting of 0.5% sodium deoxycholate, 0.1% SDS, 1% NP40, 1 mM EDTA, and 50 mM Tris (pH 8.0) for 30 minutes, followed by centrifugation to pellet the cells. The supernatant was then incubated overnight with IGF2BP3 antibody (RN009P, Medical & Biological Laboratories, Japan) or Normal Rabbit IgG (#2729, Cell Signaling Technology), and A/G magnetic beads (#P2029, Beyotime, China) were added and rotated for 2 hours. After three washings with RIPA buffer, RNA was extracted after cross-linking and subjected to RT-qPCR.
